# Identification of a unique subpopulation of mucosal fibroblasts in colorectal cancer with tumor-restraining characteristics

**DOI:** 10.1016/j.mocell.2025.100263

**Published:** 2025-08-05

**Authors:** Jamin Ku, Eunjin Jeong, Jeong-Ryeol Gong, Kwang-Hyun Cho, Chang Ohk Sung, Seok-Hyung Kim

**Affiliations:** 1 Department of Health Sciences and Technology, SAIHST, Sungkyunkwan University, Seoul 06355, Republic of Korea; 2 Department of Bio and Brain Engineering, Korea Advanced Institute of Science and Technology, Daejeon 34141, Republic of Korea; 3 Department of Pathology, Asan Medical Center, University of Ulsan College of Medicine, Seoul, Republic of Korea; 4 Department of Medical Science, Asan Medical Institute of Convergence Science and Technology, Asan Medical Center, University of Ulsan College of Medicine, Seoul, Republic of Korea; 5 Department of Pathology, Samsung Medical Center, Sungkyunkwan University School of Medicine, Seoul, Republic of Korea

**Keywords:** ADAMDEC1, Cancer-associated fibroblast, CXCL14, Single-cell sequencing, Tumor-restraining cancer-associated fibroblast

## Abstract

While tumor-restraining cancer-associated fibroblasts (Tr-CAFs) have been investigated in various cancers, their existence in colorectal cancer remains unexplored. We performed a comprehensive analysis of diverse colorectal cancer datasets, including single-cell RNA-seq/ATAC-seq data from colorectal samples, TCGA RNA-seq, and histological samples. We identified a fibroblast subpopulation uniquely expressing ADAMDEC1, CXCL14, EDNRB, and PROCR, strongly associated with favorable patient outcomes, implicating their role as Tr-CAFs. Pseudotime trajectory analysis suggested these cells as terminally differentiated mucosal fibroblasts. Pathway analysis indicated that this subpopulation was significantly associated with tumor-suppressive functions, such as reduced extracellular matrix secretion, augmented immune response, and enhanced responsiveness to immunotherapy. Single-cell ATAC-seq analysis revealed that this putative Tr-CAF subset exhibited unique epigenetic profiles characterized by superenhancer-regulated tumor-suppressive genes, thereby supporting its identity as a stable lineage rather than a transient phenotypic state induced by external stimuli. Immunohistochemistry showed that key markers identifying this putative Tr-CAF subset—CXCL14, ADAMDEC1, EDNRB, and PROCR—were predominantly localized to fibroblasts within normal colonic mucosa and less frequently in cancer-associated fibroblasts (CAFs). Their expression levels exhibited statistically significant associations with favorable clinicopathological indicators, including prolonged disease-free survival. Notably, ADAMDEC1 expression in CAFs was significantly correlated with T-cell infiltration within the tumor microenvironment. In conclusion, our investigation elucidates the characteristics and clinical relevance of Tr-CAFs in colorectal cancer, suggesting novel avenues for targeted anti-CAF therapy.

## INTRODUCTION

Cancer-associated fibroblasts (CAFs) are a crucial component of the cancer microenvironment, and they significantly impact cancer progression through various functions such as secretion and remodeling of extracellular matrix (ECM), extensive reciprocal signaling interactions with cancer cells, and immune response regulation (Sahai et al., 2020). As a result, targeting CAFs has emerged as a promising therapeutic strategy against cancer. However, modulating CAFs for therapeutic benefit presents several challenges, one of which is that CAFs can have both protumorigenic and antitumorigenic effects (Sahai et al., 2020). Therefore, the emerging consensus in recent oncological research is that an effective anti-CAF drug should specifically inhibit tumor-promoting CAFs while simultaneously either enhancing tumor-restraining cancer-associated fibroblasts (Tr-CAFs) or at the very least, not suppressing them. To achieve this, understanding and identifying tumor-repressive CAFs is imperative (Sahai et al., 2020).

A number of reports suggested the presence of Tr-CAFs in autochthonous mouse pancreatic cancer model. In a study by Özdemir et al. (2014), depleting the myofibroblast population in pancreatic ductal adenocarcinoma (PDAC) led to cancer progression with reduced animal survival due to induced immunosuppression. Furthermore, Rhim and colleagues found that reduced stromal content in a sonic hedgehog-deficient mice model of PDAC resulted in more aggressive tumors, which suggest that at least a portion of the stromal compartment, including CAFs, can restrain tumor growth (Rhim et al., 2014, Simon and Salhia, 2022). In line with these reports, in human pancreatic ductal carcinoma tissues, low intratumoral FAP+ CAF counts were reported to be significantly correlated with reduced overall survival suggesting a potential tumor-restraining role of the tumor stroma (Park et al., 2017).

Recently, Meflin-positive CAF has been reported to be a candidate for Tr-CAF in pancreatic cancer. Meflin-positive CAFs were reported to be correlated to favorable PDAC patient outcomes (Mizutani et al., 2019). Xenograft models of PDAC grown with immortalized human pancreatic stellate cells transduced with Meflin showed tumor regression and lower infiltration of protumoral α-SMA+ CAFs in mice when compared with controls (Mizutani et al., 2019). In agreement with the recent study by Tew et al, Mizutani and colleagues observed that Meflin-related tumor-inhibitory effects were linked to collagen deposition, as tumors growing in Meflin-knockout mice showed straighter and wider collagen structures as compared with tumors in wild-type mice (Mizutani et al., 2019, Tew et al., 2019).

Despite such progress in Tr-CAF research, the identity of Tr-CAFs in colorectal cancer remains unclear. To address this issue, we first identified good prognosis-associated fibroblast-specific genes, which are likely to be enriched in Tr-CAFs, and then used them to identify potential Tr-CAF subpopulations. As a result, we identified the suspected Tr-CAF subset existing naturally as terminally differentiated colonic mucosal fibroblasts enhances antitumor immunity. Our findings suggest that Tr-CAF subpopulation in colon cancer may originate from terminally differentiated colonic mucosal fibroblasts.

## MATERIALS AND METHODS

### Analysis of Single-cell RNA-seq Datasets

Public scRNA-seq datasets were used for this study; 3 colorectal cancer datasets were obtained from the Gene Expression Omnibus with GSE132465 (Lee et al., 2020), GSE144735 (Luo et al., 2022), and GSE178341 (Pelka et al., 2021) and the ArrayExpress database under accession numbers E-MTAB-8107 (Qian et al., 2020). Filtration of the raw count matrix, normalization, scaling, dimensionality reduction, cell clustering, and differential gene expression analysis was performed by Seurat package (v 4.2.2) (Hao et al., 2024) in R (v 4.2.2). We retained cells with more than 200 and fewer than 6,000 detected genes, more than 1,000 UMI counts, and filtered out cells with a mitochondrial gene ratio greater than 25%. After the filtration, we normalized the data in each sample by the “NormalizeData” function with default parameters. By this function, log (feature counts/total counts for that cell + 1) was generated. To find the top 1,000 variable genes that are used to dimensionality reduction, we used the “FindVariableFeatures” function. We set the parameters as follows: dispersion cutoff > 0.5, 0.0125 < mean cutoff < 3. Then the expression levels of genes are scaled, and the “RunPCA” function was applied on scaled data. To visualize the clustered result, we applied Uniform Manifold Approximation and Projection (UMAP) algorithm. To identify differentially expressed genes (DEGs) and robust, cell-specific markers of fibroblasts, we computed DEGs for each cell cluster using the “FindAllMarkers” and “FindMarkers” functions.

### Evaluation of Fibroblast-specific Genes for Impact on Patients’ Survival Using TCGA Colorectal Cancer Bulk Tissue RNA-seq Data and Clinical Information

Normalized gene expression data (illuminahiseq_rnaseqv2-RSEM_gene_normalized) and corresponding clinical data for colon adenocarcinoma (COAD, *n* = 274) and rectum adenocarcinoma (READ, *n* = 88) were downloaded (https://gdac.broadinstitute.org/). The survival analysis was performed using R (v 4.2.2) with the following steps:

1. Division into High- and Low-Expression Groups: Patients were divided into 2 groups based on the expression level of the target gene. The cutoff for defining high- and low- expression groups was determined using appropriate statistical methods (maxstat R package).
2. Kaplan-Meier Analysis: Kaplan-Meier survival curves were generated to visualize the survival differences between the high- and low-expression groups. The log-rank test was used to assess the statistical significance of the survival differences. Statistical significance was set at a predetermined alpha level (eg, *P* < 0.05).

### Pseudotime Analysis

The “monocle” (v 2.22.0) (Qiu et al., 2017) R package was used to construct a trajectory and find pseudotime of CAFs. The CellDataSet object was constructed by the “newCellDataSet” function from the “RNA” assay data of the Seurat object. Then we performed the “estimateSizeFacotrs” function and the “estimateDispersions” function to the CellDataSet object for further analysis. The “differentialGeneTest” function was performed to find each fibroblast-cluster-specific DEGs. Only DEGs with adjusted *P* values less than 0.001 were used to the “setOrderingFilter” function to construct a trajectory.

### Pathway Analysis and Gene Set Enrichment Analysis

Gene Set Enrichment Analysis (GSEA) was performed as follows: (1) Gene Ranking: Genes were ranked based on their differential expression statistics. A preranked gene list was generated considering the significance and direction of gene expression changes. (2) Using the GSEAplot package (v 0.1.0) (Innis et al., 2021), enrichment scores for each gene set were computed. The enrichment scores reflect the degree to which a gene set is over-represented at the top or bottom of the ranked list of genes.

Pathway Analysis was performed using ClusterProfiler (v 4.3) (Wu et al., 2021). Specifically, over-representation analysis was done to detect enriched or depleted sets of genes in favorable analysis–associated fibroblast subpopulation. Enrichment results were visualized as dot plots, bar plots, and cnetplots using functions available in the ClusterProfiler package. Finally, the results were adjusted for multiple comparisons using the Benjamini-Hochberg procedure to control the false discovery rate. Only pathways with an adjusted *P* value (q value) less than 0.05 were considered significant.

The transcription factor (TF) activity for our dataset was inferred using the Bayesian Inference Transcription Factor Activity Model (BITFAM) (Gao et al., 2021). And Cell Type Enrichment Analysis was done using “xCell” package (v 1.1.0) (Aran et al., 2017) to compare the composition of cell types in samples in these TCGA bulk RNA-seq data.

### Preparation and Single-cell RNA Sequencing of a Colon Cancer Tissue Sample

Ex vivo fibroblast culture from stage II, T3 N0 M0 colon cancer tissue was established and analyzed by single-cell RNA sequencing as described in Supplementary Material and Method.

### Random Forest Models

Random Forest models were trained in R using “randomForest” R package (version 4.7-1.1) with scaled and normalized gene expression values (see above, Seurat methods) for each gene as training features and the following parameters: ntree = 1,000; mtry = square root of total genes in each model. For Random Forest models, the data were preprocessed as follows. scRNA-seq data of cultured CAFs and scRNA-seq dataset (E-MATB-8107) were merged using “Seurat” R package (Hao et al., 2024) and batch effect between them was corrected using “harmony” R package (Korsunsky et al., 2019). Random Forest models were trained using E-MATB-8107 dataset and then applied to scRNA-seq data of cultured CAFs.

### Preparation and Analysis of Public Single-cell RNA and ATAC-seq Datasets

Single-cell RNA and ATAC-seq data from adjacent normal tissues and colorectal cancer were retrieved from the Gene Expression Omnibus (GSE201336) (Becker et al., 2022). Detailed method is described in Supplementary Material and Method.

### Immunohistochemistry and Tissue Samples

Tissue samples were prepared from patients with colorectal cancer (*n* = 300) after ethical approval by the institutional review board (SMC 2021-02-048) of Samsung Medical Center (Seoul, Korea). All patients who participated in the study signed the informed consent forms and the study protocol conforms to the ethical guidelines of the 1975 Declaration of Helsinki (sixth revision, 2008). Tissue microarray was produced and immunostainings were performed as described in Supplementary Material and Method.

### Statistical Analysis

Statistical analyses were performed using R version 4.2.1. The differences were compared using the 2-tailed t test. Correlation analysis of the continuous variables was performed using Spearman’s or Pearson's correlation analysis. A log-rank test was performed to evaluate survival differences between groups. Overall survival was determined using the Kaplan-Meier method, and analysis was performed using the R program. Survival was measured from the date of surgery, and survival curves were compared using the log-rank test.

### Ethics Declarations

The study was reviewed and approved by the institutional review board of Samsung Medical Center (Seoul, Korea) (SMC 2021-02-048).

## RESULTS

### Identification of a Fibroblast Subpopulation Associated With Favorable Prognosis as Potential Tr-CAFs in Colon Cancer

**Fig. 1:**
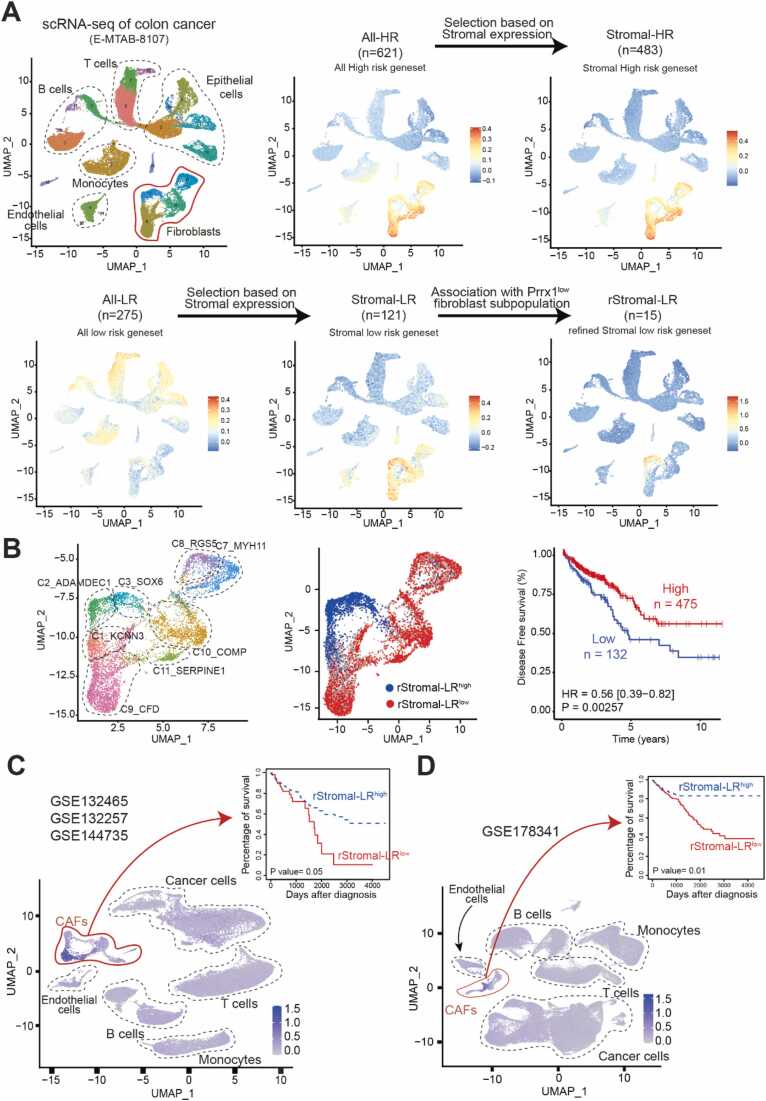
Unraveling the fibroblast subpopulation associated with favorable prognosis. (A) Fibroblast-specific gene sets associated with high and low risk (termed stromal-HR and rStromal-LR, respectively) were identified using TCGA colorectal cancer RNA-seq data and the E-MTAB-8107 scRNA-seq dataset. (B) Enrichment of rStromal-LR within a specific fibroblast subpopulation in scRNA-seq dataset (E-MATB-8107) and the correlation of rStromal-LR^high^ subpopulation with favorable prognosis of colorectal cancer. (C, D) Similar enrichments of rStromal-LR in fibroblast subpopulation from additional scRNA-seq datasets of colorectal cancer (GSE132465, GSE132257, GSE144735, and GSE178341) and their respective links of rStromal-LR^high^ subpopulation to favorable clinical outcomes.

In this study, we hypothesized that a subset of CAFs linked to favorable prognosis might represent Tr-CAFs, given that a higher prevalence of Tr-CAFs in cancer tissue could be associated with improved clinical outcomes. To explore this hypothesis, we aimed to identify a CAF subpopulation strongly associated with positive clinical outcomes. To this end, we analyzed the TCGA RNA-seq dataset of colorectal cancer (The Cancer Genome Atlas Network, 2012) and evaluated the association of each gene with disease-free survival. As a result, we identified 621 high-risk genes (All-HR; HR > 1, *P* < 0.05) and 275 low-risk genes (All-LR; HR < 1, *P* < 0.05) associated with poor prognosis (Fig. 1A, Supplementary Fig. 1A).

Next, to discover fibroblast-specific prognostic genes, we filtered these candidate genes by examining their expression patterns across different cell types at the single-cell resolution, using a colorectal cancer single-cell RNA-seq dataset (E-MTAB-8107) (Qian et al., 2020). This analysis revealed that 483 of the 621 All-HR genes and 121 of the 275 All-LR genes were specifically expressed in fibroblasts (Fig. 1A, Supplementary Fig. 1A).

To further refine the list of low-risk fibroblast genes and capture those with potential functional tumor-suppressive properties, we focused on genes enriched in the PRRX1^low^ fibroblast group compared with the PRRX1^high^ group, since PRRX1 is a master TF implicated in tumor-promoting CAF phenotypes (Lee et al., 2022, Yeo et al., 2018). As a result, 15 of the 121 Stromal-LR genes were significantly enriched in the PRRX1^low^ fibroblasts, and we termed these 15 genes as refined stromal-LR (rStromal-LR) genes (Fig. 1A, Supplementary Fig. 1A, Supplementary Table 1). We validated this rStromal-LR gene set by confirming that the colon cancer patients whose tumor tissue showed high expression of the rStromal-LR gene set had significantly improved disease-free survival (Supplementary Fig. 1E). Functional annotation indicated that these genes were primarily associated with homeostasis process during wound healing and immune modulation (Supplementary Fig. 1F).

Next, we sought to identify fibroblast subpopulation enriched for the rStromal-LR gene set. To do this, we computed the enrichment score for the rStromal-LR gene set across individual fibroblast cells in E-MTAB-8107 scRNA-seq dataset using the “AddModuleScore” function from the Seurat R package. Notably, these genes were prominently enriched within a distinct fibroblast subpopulation (Fig. 1B). This rStromal-LR^high^ subset overlapped with 2 fibroblast clusters, C2_ADAMDEC1 and C3_SOX6, as described by Qian et al. (2020). We further validated the association of rStromal-LR^high^ subset with favorable prognosis by showing that the colorectal cancer patients with high expression of rStromal-LR^high^ subset-specific genes had significantly improved disease-free survival using the TCGA database (Fig. 1B).

Additionally, we validated the rStromal-LR^high^ subset using a complementary approach focused on T stage, which reflects tumor invasion depth. We divided the TCGA colorectal cancer patients into T1/2 (low invasion) and T3/4 (high invasion) groups, then assessed fibroblast-specific low-risk genes associated with disease-free survival within each group. Interestingly, T3-/4-specific low-risk genes (*n* = 10) were also specifically enriched in the same rStromal-LR^high^ cluster (Supplementary Fig. 1G).

Our findings were consistently replicated across additional colorectal cancer single-cell RNA-seq datasets. Analysis of the dataset by Lee et al. (2020), which included 17,000 cells from 25 colorectal cancer patients, demonstrated that the rStromal-LR gene set was distinctly enriched in a specific fibroblast subpopulation that showed a strong association with improved patient outcomes (Fig. 1C). Similarly, analysis of the dataset from Pelka et al. (2021), comprising 25,000 cells from 25 colorectal cancer patients, confirmed that the rStromal-LR genes were notably enriched in a unique fibroblast subpopulation associated with favorable clinical outcomes (Fig. 1D).

Taken together, these results underscore the likelihood that this unique fibroblast subpopulation in colorectal cancer is intrinsically linked to a better prognosis.

### The Distinct Fibroblast Subpopulation Associated With Favorable Prognosis in Colorectal Cancer Is Closely Related to Terminally Differentiated Mucosal Fibroblasts of Colon

To investigate the origin and identity of this unique fibroblast population associated with a favorable prognosis, we first isolated fibroblasts from the colorectal cancer single-cell RNA-seq dataset (E-MTAB-8107) and then performed unsupervised clustering to segment them into distinct subpopulations. The resulting clusters were visualized using UMAP, revealing 4 distinct subpopulations of colon fibroblasts.

**Fig. 2:**
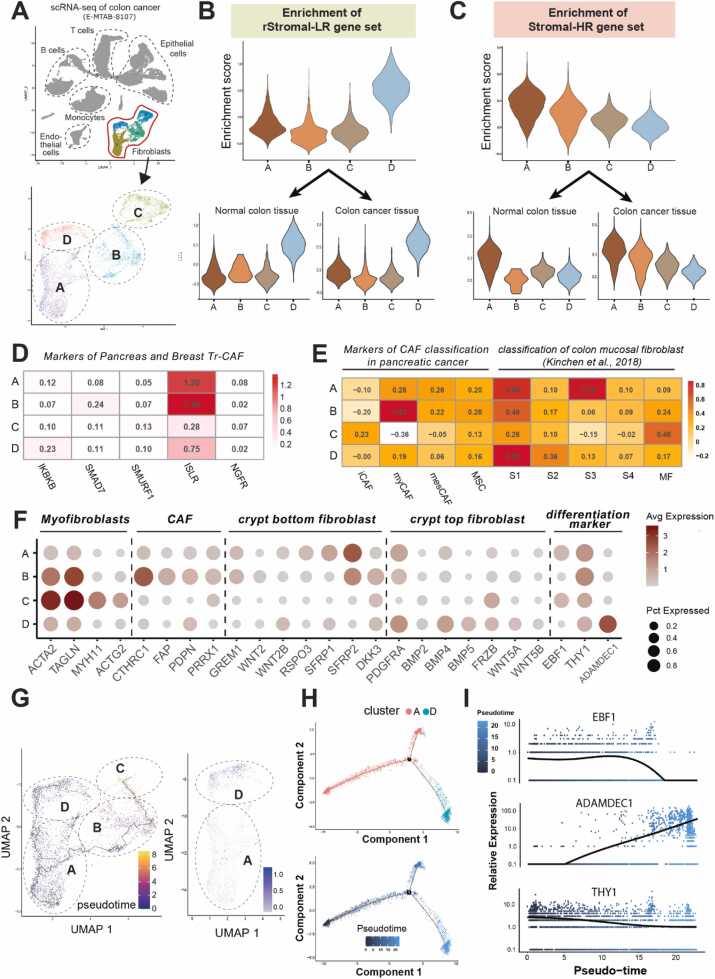
Characterization of fibroblast subtypes associated with prognosis in colorectal cancer. (A) Stratification of fibroblasts from the scRNA-seq dataset (E-MATB-8107) into 4 subpopulations. (B, C) Correlation between each fibroblast subpopulation and either low or high risk, respectively. (D) Expression of tumor-restraining CAF markers (previously identified in pancreatic and breast tumors) across stromal clusters A to D. The heatmap shows the average expression (z-score normalized) of representative Tr-CAF markers (KRKB, SMAD2, SMURF1, ISLR, and NGFR) within each stromal cluster. (E) Comparison of stromal clusters with known CAF classifications. Left: average expression of CAF subtype markers (iCAF, myCAF, mesCAF, and MSC) identified in pancreatic cancer. Right: expression patterns of stromal clusters aligned with colon mucosal fibroblast subtypes (S1-S4, MF). (F) Expression of fibroblast subtype and crypt differentiation markers across stromal clusters. (G, H) Trajectory analysis indicating that fibroblasts associated with low risk represent terminally differentiated fibroblasts. (I) Progressive shifts in the expression of crucial differentiation markers along a pseudotime gradient.

Subsequently, we evaluated these 4 fibroblast subpopulations for their enrichment of either rStromal-LR genes or Stromal-HR genes in both normal and neoplastic colon tissues (Fig. 2B, C). Notably, *cluster D* showed strong enrichment for the rStromal-LR gene set, while demonstrating no significant association with the Stromal-HR gene set. Furthermore, this enrichment pattern for *cluster D* was consistent across both normal and cancerous tissues, suggesting its inherent stability regardless of tissue context (Fig. 2B, C).

To further define the characteristics of “cluster D,” we evaluated its expression of recognized fibroblast subtype markers. Initially, we explored its expression of known Tr-CAF markers, including ISLR (Meflin), IKBKB, SMURF1, SMAD7, and NGFR. Notably, “cluster D” exhibited negligible expression for these markers (Fig. 2D). Subsequently, we assessed “cluster D” in the context of Öhlund et al’s CAF classifications (Öhlund et al., 2017), encompassing iCAF, myCAF, and mesCAF. Yet, “cluster D” did not correspond to any of these subgroups, which were primarily characterized for pancreatic cancer (Fig. 2E). Because these markers and classifications were primarily derived from pancreatic cancer studies, we shifted our focus to markers specific to colon fibroblasts.

Indeed, “cluster A” and “cluster D” prominently expressed gene sets characteristic of colonic mucosal fibroblasts (Kinchen et al., 2018) (Fig. 2F). Following Kinchen et al's classification of colonic mucosa–specific fibroblasts into types S1, S2, and S3 (Kinchen et al., 2018), “cluster D” predominantly aligned with S1 and S2. Conversely, “cluster C” emerged as a definitive myofibroblast subset, marked by elevated expression of ACTA2, TAGLN, MYH11, and ACTG2 (Fig. 2G).

In addition, we evaluated the fibroblast classification proposed by Brügger et al. (2020), which segregates colonic mucosal fibroblasts into “crypt top fibroblasts” and “crypt bottom fibroblasts.” Our assessment suggested that “cluster D,” characterized by high PDGFRA expression and reduced RSPO3 and GREM1 levels, is analogous to “crypt top fibroblasts” (Fig. 2H). Meanwhile, “cluster A,” exhibiting elevated RSPO3 and GREM1 alongside relatively lower PDGFRA expression, appears to correspond to “crypt bottom fibroblasts” (Fig. 2H).

“Cluster A” distinctively exhibited high expression of “stromal 3(S3)” gene set, previously described as a stem cell–like fibroblast subpopulation (Fig. 2F) (Kinchen et al., 2018). Furthermore, cluster “A” exhibited prominent EBF1 expression, identified as a marker for progenitor subpopulations within colonic mucosal fibroblasts (Fig. 2I) (Kinchen et al., 2018). On the other hand, “cluster D” was distinctively marked by its strong expression of “ADAMDEC1,” a marker associated with terminally differentiated colonic mucosal fibroblasts (Fig. 2I) (Kinchen et al., 2018).

Given these results, we hypothesized that “cluster D” might differentiate from “cluster A.” To substantiate this hypothesis, we undertook diffusion pseudotime analysis and elucidated the differentiation trajectory of mucosal fibroblasts. Like Kinchen et al’s study, we excluded myofibroblast (“cluster C”) from our analysis, as these cells are distinctly segregated from mucosal fibroblast subpopulation (Kinchen et al., 2018). Aligned with our hypothesis, the pseudotime analysis suggested a differentiation path initiating from “cluster A” and culminating in the terminal differentiation observed in “cluster D” (Fig. 2G, H).

### Favorably Prognostic CAF Subpopulation Likely Thwarts Tumor Progression Through Diminished ECM Secretion and Augmented Antitumor Immunity

**Fig. 3:**
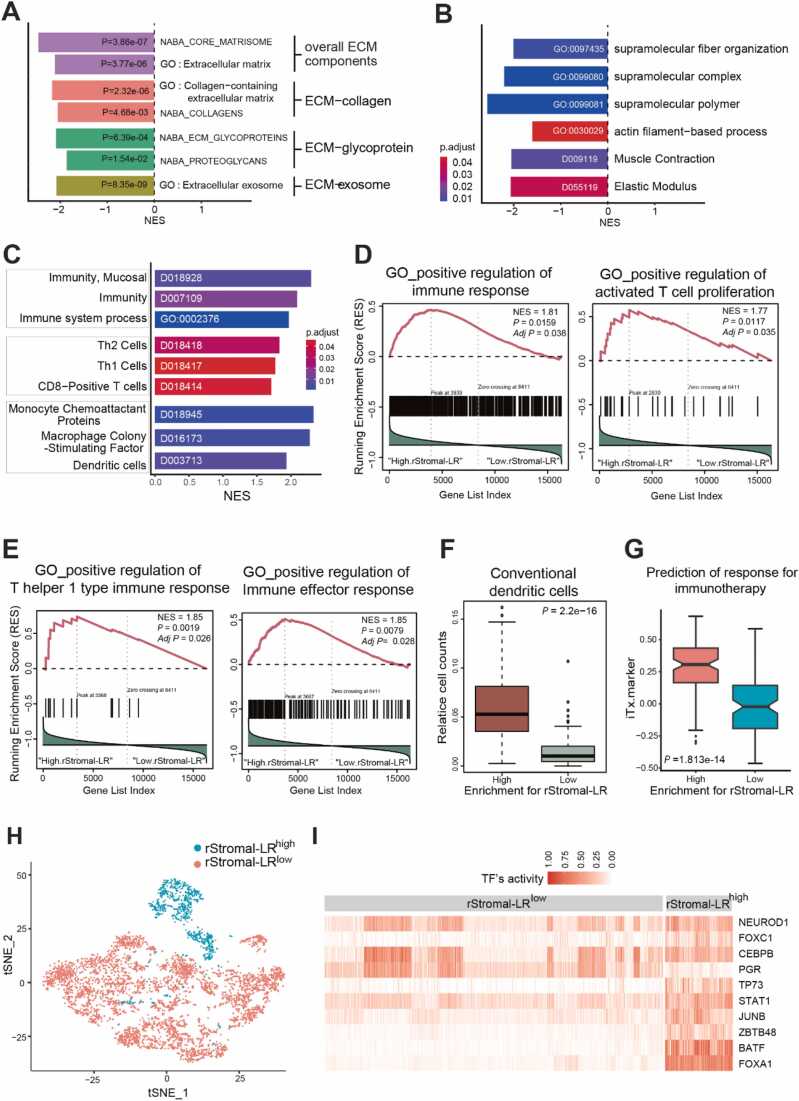
Functional ontology enriched in favorable prognosis--linked fibroblast subpopulation. (A, B) Ontology terms associated with ECM were negatively enriched in favorable prognosis–linked fibroblasts. (C) Ontology terms associated with immunity were enriched in favorable prognosis–linked fibroblasts. (D, E) Immunity gene set signature was correlated with rStromal-LR expression in TCGA colorectal cancer. (F) Enrichment of dendritic cells in rStromal-LR^high^ cohort of TCGA RNA-seq dataset of colorectal cancer. (G) Furthermore, a 13-gene signature to predict a heightened response to immunotherapy was significant in the rStromal-LR^high^ cohort of TCGA RNA-seq dataset of colorectal cancer. (H) Unsupervised clustering of fibroblasts from single-cell RNA-seq dataset (E-MTAB-8107) using BITFAM (Bayesian Inference Transcription Factor Activity model). (I) Transcription factors enriched in rStromal^high^ cluster-based analysis using BITFAM.

To elucidate the functional attributes of fibroblast subpopulation associated with favorable prognosis, we divided fibroblasts from the colorectal cancer single-cell dataset (E-MTAB-8107) into 2 groups: rStromal-LR^high^ and rStromal-LR^low^ fibroblasts. We then conducted a GSEA to reveal the active biological processes in rStromal-LR^high^ CAFs (Fig. 3A-C).

Remarkably, gene sets related to immune activation were significantly enriched in rStromal-LR^high^ CAFs compared with their rStromal-LR^low^ counterparts. In contrast, pathways related to developmental process and ECM were notably under-represented in rStromal-LR^high^ CAFs (Table 1). Specifically, gene sets concerning the secretion and organization of ECM, such as collagen and proteoglycan, were consistently downregulated in rStromal-LR^high^ CAFs (Fig. 3A, B). On the other hand, gene sets of several pathways central to immune response, including mucosal immunity and the activities of T cells, and dendritic cells, were notably elevated in rStromal-LR^high^ CAFs (Fig. 3C).

**Table 1:** GO-biological process of Tr-CAF-like subpopulations

No	ID	Description	Set size	ES	NES	*P* value	q value
1	GO:0071345	Cellular response to cytokine stimulus	106	0.392	1.931	0.00035	0.00630
2	GO:0006955	Immune response	147	0.361	1.876	0.00013	0.00489
3	GO:0002376	Immune system process	238	0.335	1.856	2.21E−05	0.00196
4	GO:0034097	Response to cytokine	119	0.372	1.849	0.00033	0.00630
5	GO:0042592	Homeostatic process	177	0.342	1.836	0.00018	0.00489
6	GO:0002682	Regulation of immune system process	149	0.343	1.780	0.00050	0.00814
7	GO:0048878	Chemical homeostasis	106	0.359	1.771	0.00204	0.02136
8	GO:0006952	Sefense response	166	0.334	1.761	0.00066	0.00904
9	GO:0044419	Biological process involved in interspecies interaction between organisms	154	0.322	1.672	0.00156	0.01829
10	GO:0051707	Response to other organism	140	0.324	1.659	0.00289	0.02576
11	GO:0032101	Regulation of response to external stimulus	131	0.326	1.658	0.00422	0.02848
12	GO:0010033	Response to organic substance	367	0.283	1.655	0.00015	0.00489
13	GO:0043207	Response to external biotic stimulus	141	0.322	1.650	0.00411	0.02848
14	GO:0014070	Response to organic cyclic compound	124	0.327	1.638	0.00375	0.02848
15	GO:0009607	Response to biotic stimulus	149	0.315	1.635	0.00339	0.02848
16	GO:0009605	Response to external stimulus	283	0.287	1.628	0.00058	0.00873
17	GO:0042221	Response to chemical	457	0.266	1.583	0.00012	0.00489
18	GO:0071310	Cellular response to organic substance	299	0.262	1.501	0.00391	0.02848
19	GO:0070887	Cellular response to chemical stimulus	376	0.253	1.485	0.00279	0.02576
20	GO:0007165	Signal transduction	497	0.242	1.453	0.00164	0.01829
21	GO:0065008	Regulation of biological quality	369	0.246	1.439	0.00432	0.02848
22	GO:0009653	Anatomical structure morphogenesis	331	−0.270	−1.612	0.00023	0.00513
23	GO:0072359	Circulatory system development	194	−0.286	−1.625	0.00110	0.01398
24	GO:0030029	Actin filament–based process	119	−0.314	−1.658	0.00406	0.02848
25	GO:0009887	Animal organ morphogenesis	144	−0.303	−1.661	0.00260	0.02573
26	GO:0009888	Tissue development	254	−0.289	−1.686	0.00019	0.00489
27	GO:0097435	Supramolecular fiber organization	131	−0.381	−2.029	1.81E−05	0.00196

ES, enrichment scores; Tr-CAF, tumor-restraining cancer-associated fibroblast.

Based on our findings, we hypothesized that rStromal-LR^high^ CAFs promote the anticancer immune response. To validate this hypothesis, we analyzed the bulk TCGA RNA-seq data from 650 colorectal cancer patients. Patients were categorized into either rStromal-LR^high^ or rStromal-LR^low^ cohorts based on the expression of rStromal-LR genes, and their RNA expression profiles were compared using GSEA. Intriguingly, the rStromal-LR^high^ group displayed significant enhancement in the overall immune response, proliferation of activated T cells, and the Th1 immune response relative to the rStromal-LR^low^ group (Fig. 3D, E). Using the xCell package (Aran et al., 2017), we further evaluated the proportion of different immune cell subtypes present within rStromal-LR^high^ cancer tissue. This revealed a pronounced enrichment of dendritic cells in rStromal-LR^high^ colorectal tumors (Fig. 3F). Additionally, a multigene signature associated with a stronger immune response to immunotherapy (Yang et al., 2022) was prominently upregulated in the rStromal-LR^high^ group (Fig. 3G).

To elucidate the regulatory framework of these rStromal-LR^high^ CAFs, we sought to identify the pivotal TFs orchestrating the cellular programming of these fibroblasts. To infer such TFs, we utilized the “Bayesian Inference Transcription Factor Activity model” (BITFAM) to analyze fibroblasts from the single-cell RNA-seq datasets (E-MTAB-8107). A distinguishing feature of BITFAM is that it leverages all published TF ChIP-seq data, thereby refining predictions of TF activities at the single-cell level (Gao et al., 2021). Upon clustering analysis based solely on transcriptional activity inferred by BITFAM, rStromal-LR^high^ CAFs clearly stood out as a unique subpopulation, reinforcing the earlier UMAP results (Fig. 3H). The further analysis using BITFAM revealed several TFs predominantly enriched in rStromal-LR^high^ CAFs, including TP73, STAT1, JUNB, ZBTB48, BATF, and FOXA1. Notably, TP73 (a structural and functional homolog of TP53), JUNB, ZBTB48, and FOXA1 have been previously documented to induce cellular senescence and inhibit proliferation of fibroblasts (Fig. 3I) (Jahn et al., 2017, Li et al., 2013, Passegué and Wagner, 2000). Moreover, STAT1 has been reported to inhibit the transformation of fibroblasts into myofibroblasts (Medley et al., 2020), while BATF was found to play a pivotal role in immune response (Schraml et al., 2009). These findings are highly consistent with our earlier GSEA results, providing additional evidence supporting the hypothesis that rStromal-LR^high^ CAFs might hinder tumor progression by reducing fibroblast ECM-related function and enhancing antitumor immune responses.

### The CAF Subpopulation Associated With Good Prognosis Was Decreased in Cancer Tissue and Rarely Observed in Ex Vivo Cultured Fibroblasts From Colon Cancer Tissue

**Fig. 4:**
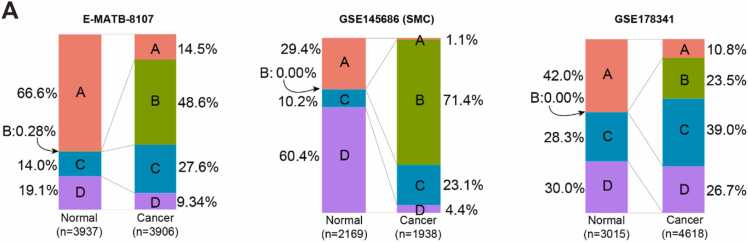
The decline of fibroblast subpopulation associated with good prognosis in cancer tissue. (A) Comparison of the proportions of fibroblast clusters such as A, B, C, and D in normal tissues against those in cancerous colorectal tissues based on 3 scRNA-seq databases: E-MATB-8107, GSE145686, and GSE178341.

During the transition from normal mucosa to cancer, significant changes in the composition of fibroblast subsets are expected to occur. To capture these changes, we compared the proportions of clusters A, B, C, and D in normal vs neoplastic colorectal tissues across 3 scRNA-seq datasets: E-MTAB-8107, GSE145686 (SMC), and GSE178341 (Fig. 4A). Notably, *cluster B* fibroblasts demonstrated a dramatic increase in cancerous tissues, while *cluster A* showed a marked decline across all datasets (Fig. 4A). This pattern suggests that *cluster B* fibroblasts are highly cancer-specific and may represent bona fide CAFs with tumor-promoting potential. We further examined the proportions of clusters A, B, C, and D across each sample within these 3 datasets. *Cluster D* fibroblasts, which represent the rStromal population, were significantly reduced in E-MTAB-8107 (*P* = 0.0231) and GSE145686 (SMC) (*P* < 0.00001), but showed only a mild, nonsignificant decrease in GSE178341 (*P* = 0.2723) (Supplementary Figs. 2-4).

We also assessed fibroblast subpopulation proportions in ex vivo CAF cultures established from stage II, T3N0M0 colon cancer tissue using standard primary CAF culture protocols. To minimize potential long-term culture biases, early-passage cells were collected and analyzed by single-cell RNA sequencing, yielding 17,000 cells. These cells predominantly expressed markers characteristic of myofibroblasts (Supplementary Fig. 5A). Using the Seurat AddModuleScore function, we computed enrichment scores for the rStromal-LR gene set across individual cells and found that only a very small fraction of cells were associated with a favorable prognosis (Supplementary Fig. 5B, C). To further quantify the distribution of clusters A to D in these ex vivo cultured CAFs, we applied a random forest classifier trained on fibroblast clusters from E-MTAB-8107. The results showed that the vast majority of cultured CAFs (>90%) were assigned to either cluster B or C, with only a small fraction corresponding to clusters A or D (Supplementary Fig. 5C). This was further supported by the rare expression of EBF1 and ADAMDEC1, while all cells remained THY1-positive, confirming their fibroblastic identity (Supplementary Fig. 5D). Collectively, these observations are consistent with the pattern of decreased clusters A and D observed in cancer tissues in the scRNA-seq data described above.

### Distinct Epigenetic Landscapes and Superenhancer-Driven Marker Genes Characterize the Tr-CAF Subpopulation

**Fig. 5:**
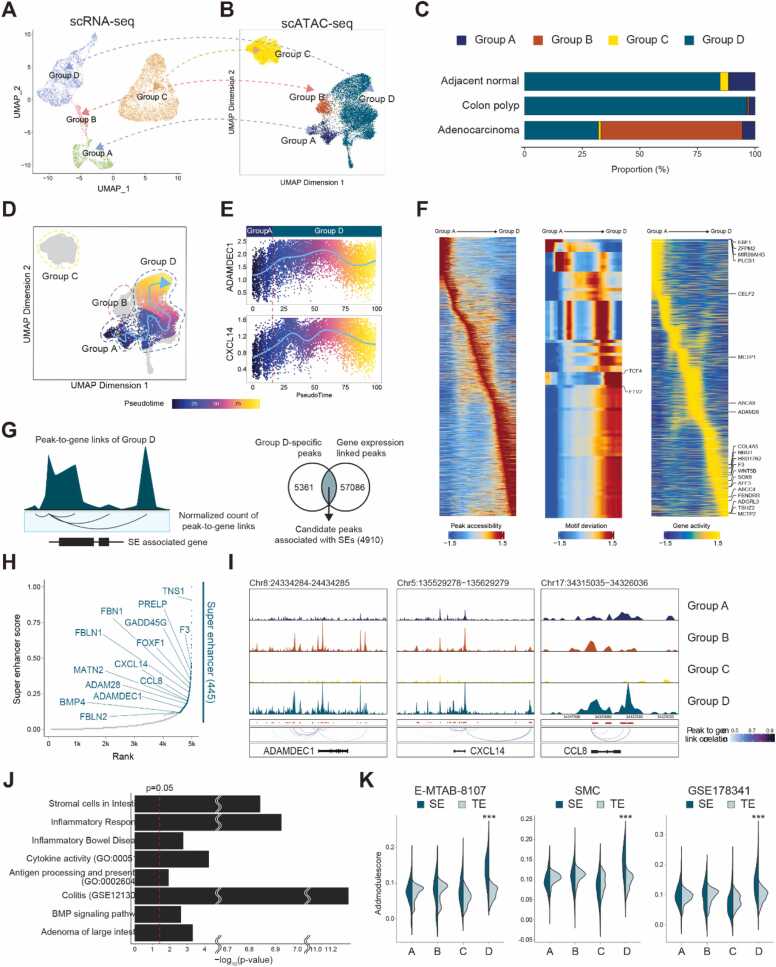
Analysis of epigenetic characteristics in Tr-CAF-like subpopulations through integrated scRNA-seq and scATAC-seq. (A) UMAP plot from scRNA-seq data displays the fibroblast subpopulations in adjacent normal colon, colon polyp, and adenocarcinoma tissues, recapitulating the subpopulations observed in Figure 2A. (B) Case-matched scATAC-seq UMAP plot confirming the 4 fibroblast subpopulations. (C) Bar graphs illustrating the distribution of fibroblast subpopulations across tissue types, showing elevated Group D fibroblast proportion in nontumor tissues and colon polyps, with a decrease in adenocarcinomas. (D) Pseudotime analysis of scATAC-seq data, indicating the likely origin of Group D fibroblasts from Group A fibroblasts. (E) Peak profiles revealing increasing chromatin accessibility of Group D marker genes (ADAMDEC1 and CXCL14) as Group A differentiates into Group D. (F) Heatmaps showing dynamic peaks (left), motif deviations (middle), and gene activity scores (right) throughout the pseudotime trajectory. (G) Schematic figure of the Tr-CAF-specific superenhancer (SE)-associated genes identification. (H) Hockey plot showing SE-associated genes. (I) Loci of ADAMDEC1, CXCL14, and CCL8 genes illustrating pseudo-bulk ATAC-seq signals from Tr-CAF subpopulation, accompanied by peak-to-gene linkages. (J) Bar plot illustrating the GO terms associated with the SE-driven genes specific to Tr-CAF subpopulation. (K) Violin plots illustrating the AddModuleScores of SE- and TE-driven genes in fibroblast subpopulations in 3 datasets.TE, typical enhancer.

To determine whether the Tr-CAF subpopulation represents a differentiated lineage or merely a transient state in response to external stimulus, we examined their epigenetic landscape. We utilized an integrated single-cell ATAC sequencing (scATAC-seq) and single-cell RNA sequencing (scRNA-seq) dataset of adjacent normal colon, colon polyp, and adenocarcinoma tissues from 15 colon cancer patients. These datasets were provided by Stanford University under the identifier GSE201336 (Becker et al., 2022). Upon initial analysis, we found that the fibroblast population, as identified through scRNA-seq, could be categorized into the same 4 distinct subpopulations as described earlier in Figure 2A (Fig. 5A).

Importantly, subsequent case-matched scATAC-seq analysis confirmed the presence of these subpopulations, including Group D fibroblasts, which are suspected to be Tr-CAF (Fig. 5B and Supplementary Fig. 6A-D). We noticed that the proportion of Group D fibroblasts was significantly elevated in nontumor tissues and colon polyps, but declined in adenocarcinomas, in accord with our earlier findings shown in Figure 4A (Fig. 5C).

Pseudotime analysis using peak profiles derived from scATAC-seq dataset also indicates that Group D fibroblasts likely originate from Group A fibroblasts, consistent with results of our previous trajectory analysis using scRNA-seq dataset (Fig. 5D). Further analysis of these peak profiles against pseudotime order revealed that chromatin accessibility peaks of marker genes for Group D fibroblasts, namely ADAMDEC1 and CXCL14, tend to increase as Group A fibroblasts differentiate into Group D (Fig. 5E). A gradual transition between these groups is evident across several metrics, including peak accessibility, TF–binding motif availability, and gene activity, as revealed through integrated analysis of both scATAC-seq and scRNA-seq data (Fig. 5F).

We next aimed to identify genes and markers specific to the Tr-CAF subpopulation that are driven by superenhancers (SEs), utilizing this epigenetic framework. To accomplish this, we developed a new analytical pipeline that combines tools from both scATAC and H3K27ac-Chromatin Immunoprecipitation sequencing (ChIP-seq). This pipeline involves 2 main steps: Step 1: Identifying key chromatin accessibility peaks that are crucial for the Tr-CAF subpopulation, Step 2: Selecting genes driven by SEs, based on the profile of these key peaks.

Specifically, we chose chromatin accessibility peaks critical for Tr-CAF using 2 criteria: first, the peak must be unique to Group D, and second, it must be significantly associated with the gene expression. To identify such peaks, we performed a peak-to-gene linkage analysis using the ArchR R package (Granja et al., 2021) (Fig. 5G). Subsequently, we ranked genes according to the number of these Tr-CAF-specific peaks selected through double criteria. Intriguingly, when plotted, these peaks showed an extremely skewed distribution; therefore, they followed an “elbow”-shaped curve like SE curve (Fig. 5H). According to a method employed to identify SE (Hnisz et al., 2013, Whyte et al., 2013), we defined SE-driven genes as those whose peak intensity lies above the curve's inflection point. In contrast, genes with peak intensities below this point are driven by typical enhancers (TEs) (Fig. 5H).

These SE-driven genes in the Tr-CAF subpopulation include not only markers specific to Group D fibroblasts such as ADAMDEC1, CXCL14, and CCL8, but also tumor suppressor genes such as EMID1, PRELP, FENDRR, and GADD45G (Chiavarina et al., 2022, Guo et al., 2021, Karpus et al., 2019, Senavirathna et al., 2021). This supports the notion that Group D fibroblasts play a role in suppressing tumors (Fig. 5I). Moreover, these SE-driven genes were predominantly involved in biological processes related to intestinal stromal cells, inflammation, antigen presentation, and the BMP signaling pathway (Fig. 5J).

To further validate the importance of SE-driven genes compared with TE-driven genes, we performed an enrichment analysis across 3 previously utilized scRNA-seq datasets. Specifically, we calculated enrichment scores for both SE- and TE-driven genes in fibroblast subpopulations using the “AddModuleScore” function. Consistent with our expectations, SE-driven genes showed higher enrichment scores in Group D fibroblasts compared with TE-driven genes. Interestingly, this pattern was unique to Group D and was not observed in other fibroblast subpopulations (Fig. 5K).

### Representative Markers of Tr-CAF Subpopulation, Including CXCL14, ADAMDEC1, EDNRB, and PROCR, Are Expressed in Colonic Mucosal Fibroblasts and Cancer-associated Fibroblasts

To validate the existence of Tr-CAF subpopulations in the histological landscape of colorectal cancer tissues, we examined the spatial expression patterns of key Tr-CAF markers. Specifically, we analyzed the expression of 4 selected markers—CXCL14, ADAMDEC1 (A Disintegrin and Metalloprotease-Like Decysin), EDNRB (Endothelin Receptor Type B), and PROCR1 (Protein C Receptor)—in 250 tissue samples that spanned normal, adenomatous, and cancerous colon tissues using antibodies to these markers and immunohistochemistry (IHC).

**Fig. 6:**
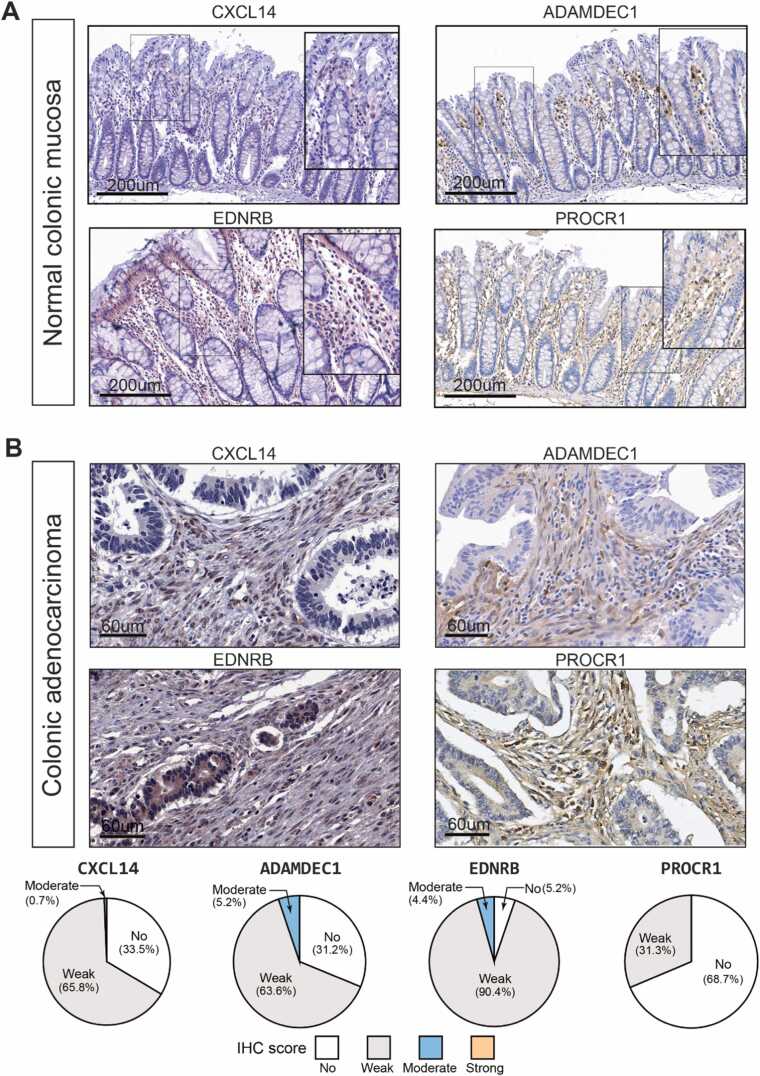
Confirmation of Tr-CAF markers in normal colonic mucosal fibroblast and cancer-associated fibroblast. (A) Immunohistochemical staining of ADAMDEC1, CXCL14, PROCR, and EDNRB was performed using samples from both normal colonic mucosa and colonic adenocarcinoma tissues. (B) The pie charts illustrate the distribution of IHC scores for each marker in the colonic tissue samples. IHC scores were categorized into 4 groups: No, Weak, Moderate, and Strong.

Based on our prior findings from scRNA-seq analyses, as shown in Figure 2F, we hypothesized that these markers of Tr-CAFs would be specifically expressed in the colonic mucosa, given that Tr-CAFs are suggested to represent terminally differentiated mucosal fibroblasts. Indeed, the 4 markers were predominantly localized in fibroblasts within the lamina propria of the normal colonic mucosa. The only exception was EDNRB, which was also observed in vascular endothelial cells (Fig. 6A). Interestingly, ADAMDEC1 was most abundant in the upper regions of the lamina propria, indicating its potential role in more advanced fibroblast differentiation.

In the majority of colorectal cancer (adenocarcinoma) tissue samples, fibroblasts surrounding tumor cells displayed either no or weak IHC staining for the 4 markers in 90.4% to 100% of cases, with only a few exceptions (Fig. 6B). Notably, the expression levels of EDNRB, PROCR1, and ADAMDEC1 were significantly reduced in CAFs compared with those in normal colonic mucosa (Supplementary Fig. 7B). Similarly, in adenoma tissues, most cases showed no or weak staining for these 4 markers in fibroblasts surrounding the tumor cells (Supplementary Fig. 7A).

### CAFs Expressing CXCL14, ADAMDEC1, EDNRB, and PROCR Are Significantly Linked to Favorable Prognosis in Colon Cancer

To evaluate whether CAFs expressing Tr-CAF markers could inhibit tumor progression, we investigated the correlation between the CAF-specific immunohistochemical (IHC) staining levels for these 4 CAF markers; CXCL14, ADAMDEC1, EDNRB, and PROCR, and the clinical outcomes of colorectal cancer patients.

**Fig. 7:**
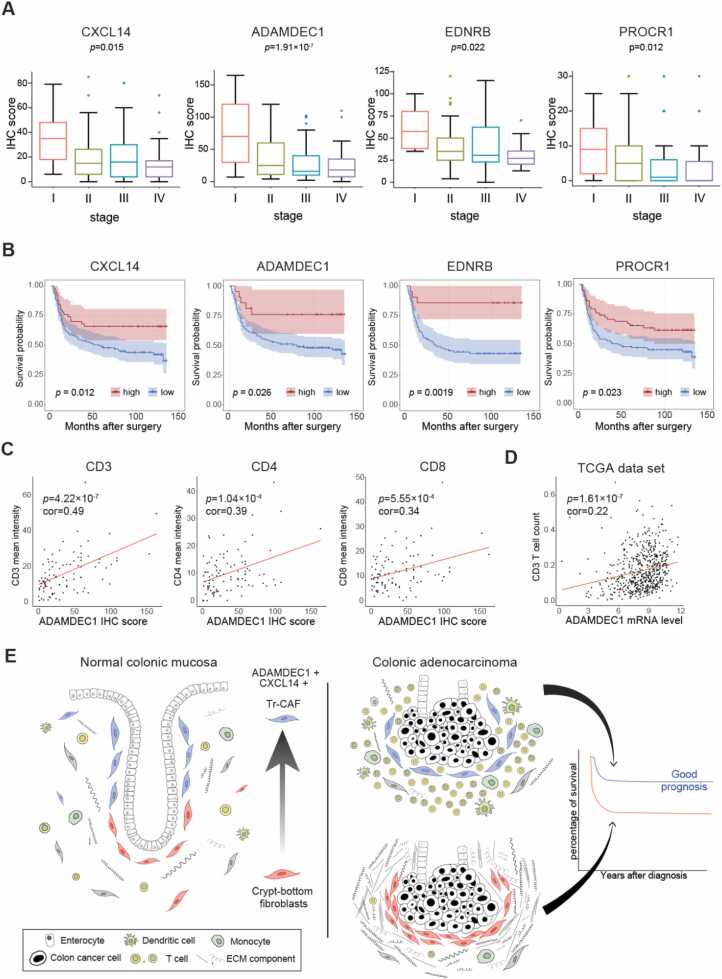
Survival rates of high Tr-CAF marker-expressing patients and their correlation with immune cells. (A) The box plots illustrating the IHC scores of CXCL14, ADAMDEC1, EDNRB, and PROCR for different cancer stages. (B) Kaplan-Meier survival curves based on the expression levels of CXCL14, ADAMDEC1, EDNRB, and PROCR in patients. (C) The scatterplot illustrates the relationship between ADAMDEC1's mean staining intensity and the mean staining intensities of CD3, CD4, and CD8 in colon cancer tissue. (D) The scatterplot illustrates the relationship between the expression mRNA level of the ADAMDEC1 and the CD3 T-cell count in colon cancer tissue from the TCGA dataset. (E) The schematic diagram illustrates the summary of our study.

Initially, we identified significant negative correlations between CAF-specific IHC scores for these 4 Tr-CAF markers and clinical stage of colorectal cancer. Specifically, a statistically significant decline in IHC scores for all 4 Tr-CAF markers was evident in advanced-stage cancers compared with early-stage cases (Fig. 7A). In addition, CAF-specific ADAMDEC1 IHC score was inversely correlated with multiple unfavorable clinicopathological parameters, such as increased tumor invasion depth, nodal and distant metastases, poor cell differentiation, perineural invasion, and increased frequency of tumor budding (Table 2). Intriguingly, the CAF-specific IHC scores for CXCL14, ADAMDEC1, and EDNRB were significantly lower in tissues from older patients (Table 2, Table 3, Table 4, Table 5).

**Table 2:** Relationships between the expression of ADAMDEC1 and clinicopathological parameters

	*Age*				
	<70 y (*n* = 157)		≥70 y (*n* = 16)		*P* value
IHC score	36.1±34.6		22.8±15.9		0.010
				
	*Gender*				
	Female		Male		*P* value
IHC score	32.6±32.6		36.7±34.0		0.424
				
	*Stage*				
	I	II	III	IV	*P* value
IHC score	73.8±53.1	39.8±32.1	25.6±25.0	27.6±29.3	0.000
		
	*Differentiation of cancer cells*				
	W/D	M/D		P/D	*P* value
IHC score	49.4±45.0	34.4±31.5		20.5±23.8	0.021
		
	*Depth of tumor invasion (T stage)*				
	1	2	3	4	*P* value
IHC score	83.0±61.7	52.7±46.3	32.7±29.5	19.7±30.0	0.001
		
	*Nodal metastasis (N stage)*				
	Absent	Low (1-3)		High (≥4)	*P* value
IHC score	44.8±38.1	27.2±24.3		25.6±27.9	0.001
		
	*Distant metastasis*				
	Negative		Positive		*P* value
IHC score	38.1±35.0		24.0±26.4		0.037
		
	*Perineural tumor invasion*				
	Negative		Positive		*P* value
IHC score	37.8±34.8		17.1±14.1		0.000
		
	*Tumor budding*				
	Absent	Low (5-9/10HPF)		High	*P* value
IHC score	41.1±34.4	33.1±29.9		19.2±24.4	0.027

IHC, immunohistochemistry.

**Table 3:** Relationships between the expression of CXCL14 and clinicopathological parameters

	*Age*				
	<75 y (*n* = 151)		≥75 y (*n* = 7)		*P* value
IHC score	21.3±20.3		8.4±6.0		0.000
				
	*Gender*				
	Female		Male		*P* value
IHC score	19.6±18.2		21.7±21.5		0.513
				
	*Stage*				
	I	II	III	IV	*P* value
IHC score	32.4±21.2	20.4±18.8	19.0±20.6	18.2±19.0	0.0309
		
	*Differentiation of cancer cells*				
	W/D	M/D		P/D	*P* value
IHC score	25.9±21.5	21.0±20.9		18.7±15.2	0.2786
		
	*Depth of tumor invasion (T stage)*				
	1	2	3	4	*P* value
IHC score	32.5±21.0	29.0±19.8	20.6±20.6	12.6±14.8	0.0076
		
	*Nodal metastasis (N stage)*				
	Absent	Low (1-3)		High (≥4)	*P* value
IHC score	21.8±19.4	20.9±23.7		18.4±17.8	0.2318
		
	*Distant metastasis*				
	Negative		Positive		*P* value
IHC score	22.6±21.0		16.3±17.3		0.151
		
	*Perineural tumor invasion*				
	Negative		Positive		*P* value
IHC score	23.0±21.5		15.4±14.5		0.251
		
	*Tumor budding*				
	Absent	Low (5-9/10HPF)		High	*P* value
IHC score	23.2±19.4	25.4±21.8		12.2±12.8	0.179

IHC, immunohistochemistry.

**Table 4:** Relationships between the expression of EDNRB and clinicopathological parameters

	*Age*				
	<52 y (*n* = 84)		≥52 y (*n* = 30)		*P* value
IHC score	50.1±28.6		38.2±24.6		0.032
				
	*Gender*				
	Female		Male		*P* value
IHC score	41.3±26.2		41.4±26.3		0.990
				
	*Stage*				
	I	II	III	IV	*P* value
IHC score	59.6±25.1	41.1±26.0	41.4±27.8	31.6±16.2	0.0344
		
	*Differentiation of cancer cells*				
	W/D	M/D		P/D	*P* value
IHC score	51.3±28.9	40.8±26.8		38.2±20.8	0.1846
		
	*Depth of tumor invasion (T stage)*				
	1	2	3	4	*P* value
IHC score	62.0±33.6	57.2±24.8	39.2±25.7	39.0±32.7	0.0054
		
	*Nodal metastasis (N stage)*				
	Absent or low (1-3)			High (≥4)	*P* value
IHC score	44.2±27.9			33.1±18.1	0.016
		
	*Distant metastasis*				
	Negative		Positive		*P* value
IHC score	44.5±28.0		30.4±14.4		0.003
		
	*Perineural tumor invasion*				
	Negative		Positive		*P* value
IHC score	40.0±24.5		32.3±19.0		0.342
		
	*Tumor budding*				
	Absent	Positive [low (5–9/10HPF) or high]			*P* value
IHC score	44.7±27.2	33.2±21.7			0.032

IHC, immunohistochemistry.

**Table 5:** Relationships between the expression of PROCR and clinicopathological parameters

	*Age*				
	<65 y (*n* = 134)		≥65 y (*n* = 47)		*P* value
IHC score	5.4±7.7		8.1±10.5		0.110
				
	*Gender*				
	Female		Male		*P* value
IHC score	6.4±9.5		5.9±7.7		0.667
	*Stage*				
	I	II	III	IV	*P* value
IHC score	9.6±8.5	7.5±9.2	4.7±8.2	5.2±7.0	0.0058
		
	*Differentiation of cancer cells*				
	W/D	M/D		P/D	*P* value
IHC score	6.8±7.7	6.0±8.4		7.4±12.6	0.779
		
	*Depth of tumor invasion (T stage)*				
	1	2	3	4	*P* value
IHC score	6.8±9.1	13.1±12.8	5.4±7.6	5.5±10.5	0.005
		
	*Nodal metastasis (N stage)*				
	Absent	Low (1-3)		High (≥4)	*P* value
IHC score	7.5±9.0	5.3±8.5		4.5±7.5	0.0153
		
	*Distant metastasis*				
	Negative		Positive		*P* value
IHC score	6.6±8.9		4.9±7.6		0.320
		
	*Perineural tumor invasion*				
	Negative		Positive		*P* value
IHC score	6.3±8.9		6.5±11.6		0.789
		
	*Tumor budding*				
	Absent	Low (5-9/10HPF)		High	*P* value
IHC score	7.1±8.6	6.4±11.0		5.4±7.5	0.3143

IHC, immunohistochemistry.

Subsequently, we investigated the association between the IHC scores of these Tr-CAF markers and patient survival rates. High CAF-specific IHC scores for each of the 4 markers were strongly correlated with favorable clinical outcomes that are in accord with our analysis from TCGA bulk RNA-seq data (Fig. 7B). When we further stratified patients into early-stage and late-stage cancer groups for additional analysis, we observed that high CXCL14 IHC score in CAFs was particularly associated with a favorable prognosis in advanced stages (Supplementary Fig. 8A). On the other hand, high ADAMDEC1 IHC score in CAFs correlated with favorable outcomes in early-stage colorectal cancer. Notably, both EDNRB and PROCR IHC scores were significantly linked to good prognoses in both early and advanced stages of the disease (Supplementary Fig. 8A).

Given the proposed role of Tr-CAFs in enhancing immune responses as depicted in Figure 3C, we examined the correlation between CAFs expressing Tr-CAF markers and number of T cells in colorectal cancer tissues. For this analysis, T cells were identified using antibodies to CD3, CD4, and CD8 through IHC on the same sets used for CAF marker staining. As anticipated, cancer tissues rich in ADAMDEC1-positive CAFs also contained significantly higher counts of CD3(+), CD4(+), and CD8(+) T cells (Fig. 7C and Supplementary Fig. 8B). A positive correlation between ADAMDEC1 mRNA level and T-cell count was again observed in the TCGA transcriptome data (Fig. 7D). These findings support the notion that the tumor-suppressive function of ADAMDEC1-positive CAFs may be facilitated through the enhancement of T-cell immunity. A comprehensive illustrative summary is presented in Figure 7E.

## DISCUSSION

Based on comprehensive analyses of scRNA-seq and scATAC-seq datasets of colorectal cancer tissues, as well as histological examinations of suspected Tr-CAF markers, our study uncovered a distinct subpopulation of fibroblasts and CAFs with potential tumor-suppressive capabilities.

Initially, we serendipitously discovered a unique fibroblast subpopulation strongly associated with favorable colon cancer prognosis. Subsequent investigations revealed that this subpopulation is likely composed of terminally differentiated mucosal fibroblasts commonly found in normal colon mucosa but present at reduced levels in cancerous tissue. Our data indicate that this favorable subpopulation, termed “Group D,” likely differentiates from a more progenitor-like “Group A” subpopulation characterized by EBF1. This is consistent with prior research indicating that “group A”–like PDGFRa^low^ fibroblast subpopulation supports the maintenance of epithelial stemness, while “group D”–like PDGFRa^high^ subpopulation promotes epithelial differentiation (Brügger et al., 2020, Kinchen et al., 2018, Li et al., 2017). Moreover, our findings further revealed that such “group D” fibroblast subpopulation could enhance anticancer immunity and exhibited diminished ECM-remodeling activity, suggesting their role as Tr-CAFs.

Importantly, our study underscores that only a subset of so-called “normal fibroblasts” within the colonic mucosa may possess tumor-repressive capabilities. And the persistent presence of such Tr-CAF subpopulation in the colonic mucosa may explain the rarity of metastasis to the colon from other primary cancer sites (Galanopoulos et al., 2018).

While Tr-CAFs are documented in other cancer types, including lung (Pellinen et al., 2023), pancreas (Djurec et al., 2018, Mizutani et al., 2019, Özdemir et al., 2014, Rhim et al., 2014), and breast (Chang et al., 2012), their presence in colorectal cancer remains underexplored. When compared with suspected Tr-CAFs in other types of cancer, the high level of PDGFRa was a common feature; however, most other known markers, including Meflin, were not significantly expressed in Tr-CAF of colorectal cancer. Considering the microscopic architecture and functions of the colonic mucosa being completely different from those of other organs, Tr-CAFs of colorectal cancer could be different from other cancer because Tr-CAF is derived from fibroblast subpopulation maintaining the homeostasis of these organs. Large-scale pan-cancer scRNA-seq analyses further validate the tissue-specific nature of both epithelial cells and fibroblasts.

Of the 4 Tr-CAF markers, CXCL14 was reported to promote the mobilization of immune cells, particularly monocytes and dendritic cells (Shurin et al., 2005). This is highly consistent with our findings of augmented dendritic cell presence in colorectal cancer tissues marked by Tr-CAF-related genes. Additionally, consistent with our findings, low levels of CXCL14 in bulk cancer tissue were reported to be linked to poor survival rates in colon cancer (Cao et al., 2013).

ADAMDEC1 was documented to be abundantly and almost exclusively expressed in subepithelial PDGFRa^high^ fibroblast within the mucosa of gastrointestinal tract (Ha et al., 2022). And studies on tissues from Crohn's disease patients indicate that ADAMDEC1 enhances mucosal innate immunity and plays a crucial role in maintaining mucosal tissue homeostasis (Ha et al., 2022, Jasso et al., 2022). Additionally, a study using ADAMDEC1 knockout mice confirmed that ADAMDEC1 is crucial in restoring mucosal tissue to its normal shape in response to damage (Ha et al., 2022). All of these reports align well with our findings, suggesting ADAMDEC1 being a marker of terminally differentiated mucosal fibroblasts as a Tr-CAF.

EDNRB (Endothelin Receptor Type B) and PROCR (Protein C Receptor) were also reported to be specifically expressed in crypt top fibroblasts of the colonic mucosa (Jasso et al., 2022, Kim et al., 2020), which reinforces our findings.

In summary, we identified a Tr-CAF subset in colorectal cancer, existing naturally as terminally differentiated mucosal fibroblasts that enhance antitumor immunity. Our finding could play a pivotal role for targeted anti-CAF drug development and reprogramming of tumor-promoting CAF to Tr-CAF.

## Author Contributions

**Jeong-Ryeol Gong:** Investigation, Validation, Visualization, Writing – review & editing. **Jamin Ku:** Investigation, Visualization, Writing – original draft, Writing – review & editing. **Eunjin Jeong:** Investigation, Visualization, Writing – original draft, Writing – review & editing. **Kwang-Hyun Cho:** Methodology, Writing – review & editing. **Chang Ohk Sung:** Methodology, Writing – review & editing. **Seok-Hyung Kim:** Conceptualization.

## Declaration of Competing Interests

The authors declare that they have no known competing financial interests or personal relationships that could have appeared to influence the work reported in this paper.

## Data Availability

All sequencing data used in this study were available at public domain in the Gene Expression Omnibus with accession numbers (GSE132465, GSE144735, GSE178341, GSE201336, GSE145686, GSE178341, and GSE201336), ArrayExpress (E-MTAB-8107).

## Appendix A Supplemental material

Supplementary material and method

## Footnotes

Appendix A Supplemental material associated with this article can be found online at doi:10.1016/j.mocell.2025.100263.
