# Supplementary material for: Identification of a unique subpopulation of mucosal fibroblasts in colorectal cancer with tumor-restraining characteristics
Source: Mol Cells. 2025 Aug 5;48(10):100263. doi: 10.1016/j.mocell.2025.100263 (PMC12419087; doi:10.1016/j.mocell.2025.100263)
Supplement: Supplementary file 1 — Supplementary material and method [file mmc1.docx]

**Supplementary Material & Method**

**Single-cell RNA-seq sample preparation, library preparation and sequencing**

CAFs were isolated from surgically resected samples from patients with colorectal cancer and maintained in Dulbecco’s modified Eagle’s medium (HyClone Laboratories) supplemented with 10% FBS, 1% penicillin, and streptomycin. We used CAFs within six passages to avoid potential senescence-associated phenotypic changes. The use of CAFs was approved by the Institutional Review Board of Samsung medical center (AJIRB-BMR-SMP-20-222). For scRNA-seq, the 10x Genomics Chromium platform was used to capture and barcode the cells to generate single-cell Gel Beads-in-Emulsion (GEMs), according to the manufacturer’s protocol. Briefly, along with the reverse transcription master mix, cell suspensions were loaded onto 10x Genomics Single Cell 30 Chips. During this step, the cells were partitioned into GEMs, along with gel beads coated with oligonucleotides. These oligonucleotides enable mRNA capture inside the droplets by 30 bp oligo-dT after cell lysis and provide barcodes to index cells (16 bp) and transcripts (12 bp unique molecular identifiers (UMI)). Following reverse transcription, cDNAs with both barcodes were amplified, and a library was constructed using the Single Cell 3´ Reagent Kit (v3.1 chemistry) for each sample. The resulting libraries were sequenced on an Illumina NovaSeq 6000 System in 2 × 150 bp paired-end mode.

**Sample demultiplexing, barcode processing, and UMI counting**

We performed sample demultiplexing, barcode processing, and UMI counting using the official 10x Genomics pipeline Cell Ranger (v6.1.1) (https://support.10xgenomics.com). Briefly, raw base call files generated by Illumina sequencers were demultiplexed into reads in the FASTQ format using bcl2fastq developed by Illumina (https://github.com/brwnj/bcl2fastq). The reads from each library were then processed separately using the ‘cellranger count’ pipeline to generate a gene-barcode matrix for each library. During this step, reads were aligned to the human reference genome (GRCh38). Cell barcodes and UMIs associated with the aligned reads were subjected to correction and filtering, and the count matrix data were pre-processed using the Seurat R package (v4.1.1). The scRNA-seq data have been deposited in the Gene Expression Omnibus (GEO) with accession No. GSE223858.

**Preparation of public single-cell RNA and ATAC-seq datasets**

Single-cell RNA and ATAC-seq data from adjacent normal tissues and colorectal cancer were retrieved from the Gene Expression Omnibus (GEO, GSE201336). Subsequently, we pre-processed the data in accordance with the guidelines provided by the authors using the ArchR packages (<https://github.com/winstonbecker/scCRC_continuum>) (31).

**Integrated scATAC and scRNA-seq Analysis**

Single-cell ATAC and RNA sequencing data were integrated using the ArchR 'addGeneIntegrationMatrix' function. This integration employed the scRNA-seq object in conjunction with either the complete scATAC-seq object or the 'core' clusters. “Peak-to-gene links” (PGLs) were determined using correlations between peak accessibility and integrated scRNA-seq expression data. This was executed with the 'addPeak2GeneLinks' function, using parameters: maxDist = 400000, corCutOff = 0.75, k = 150, overlapCutOff = 0.8, predictionCutOff = 0.35, and knnIterations = 500.

**Pseudotime Trajectory Analysis using Integrated scATAC and scRNA-seq Analysis**

To investigate the dynamics of peak accessibility, transcription factor activity, and gene activity along pseudo-time analysis was employed using the Monocle implementation within ArchR (31). The analysis's ArchR object was processed using “getTrajectories,” followed by “addTrajectory” with a predefined trajectory order: From Group A to Cluster D. Peak accessibility, ChromVAR deviation scores, and gene activity scores were then correlated throughout the pseudotime. This was achieved using the 'getTrajectory' and 'plotTrajectoryHeatmap' functions, adhering to standard parameters for gene activities and peak accessibility, and specifying "varCutOff = 0.8" for the motif matrix.

**Super Enhancer Analysis using Integrated scATAC and scRNA-seq Analysis**

For the ArchR object, PGLs were computed. Subsequently, high-confidence PGLs that overlapped with marker peaks of the core clusters were isolated, adhering to the following criteria: corCutOff = 0.35, FDR <= 1e-04, VarQATAC >= 0.25, and VarQRNA >= 0.25. The count of marker peaks associated with each gene through PGLs was then calculated and scaled to a maximum value of 1. The number of linked peaks for each gene was ranked, and the "elbow" point was identified using the ' find_curve_elbow' function from the R package pathviewr. (<https://github.com/Jeong-RyeolGong/ATACtoSE.git>)

**Immunohistochemistry and tissue samples.**

Tissue microarrays (TMA) were produced with samples with clinical data including age, sex, tumor size, depth of invasion (T), nodal status (N), metastasis (M), overall survival (OS) and disease-free survival (DFS). Staging based on TNM classification was applied according to guidelines from the 2010 American Joint Committee on Cancer staging manual. Two pathologists (COS and SHK) evaluated immunohistochemical results with no prior knowledge of clinicopathological results, and discussed any discrepancies in scores until a consensus was reached. Sections were deparaffinized with histoclear, then rehydrated with an alcohol, and finally hydrogen peroxide in methanol. The sections were then treated with TE buffer (Tris 10 mM and EDTA 1 mM, pH9.0) for antigen retrieval. Each of sections were incubated with primary antibodies: anti-ADAMDEC1 (Proteintech17833-1-AP, 1:200), anti-EDNRB (Abcam ab117529, 1:200), anti-PROCR (LSbio LS-C339622, 1:150), anti-CXCL14 (LSbio LS-B6967, 1:100), anti-CD3 (DaKo M7254, 1:100), anti-CD4 (Dako M7310 1:50), anti-CD8 (Dako M7103 1:100), antibody in TBST containing 5% skim milk for overnight at 4℃. Following three washes with PBS buffer, sections were then incubated with a Rabbit anti-mouse IgG (Jackson Immuno Research 315-005-003) or Mouse anti-rabbit IgG (Jackson Immuno Research 211-005-109) 30min at room temperature to amplify the signal. And then incubated with an anti-mouse/rabbit-specific protein kit (Envision Plus, Dako, Carpinteria, CA, USA) at room temperature, followed by peroxidase substrate ImmPACT NovaRED (Vector, Burlingame, CA, USA)) or 3,3’-diaminobenzidine tetrahydrochloride salt (DAB, Envision Plus, Dako, Carpinteria, CA, USA). We counterstained sections with Meyer’s hematoxylin and generated the virtual slide images using Aperio® AT2 virtual slide scanner (Leica, Wetzler, Germany). We measured Immunohistochemical scores for each protein expression semi-quantitatively based on staining intensity and positive cell number, assigning scores ranging from 1 to 3. Subsequently, we multiplied the intensity and percentages of positive cells. The maximum points achievable is 300.

**Correlation analysis between gene expression and immune cell infiltration with TCGA data**

To assess if ADAMDEC1 expression correlates with the infiltration of group D fibroblasts, we used transcriptome data from TCGA. We downloaded TCGA transcriptome data from the GDC portal (https://portal.gdc.cancer.gov/). The correlation between ADAMDEC1 expression in the downloaded transcriptome data was analyzed using R (Version 4.3). The analyzed results were visualized using ggplot2.
