# Supplementary material for: Identification of a unique subpopulation of mucosal fibroblasts in colorectal cancer with tumor-restraining characteristics
Source: Mol Cells. 2025 Aug 5;48(10):100263. doi: 10.1016/j.mocell.2025.100263 (PMC12419087; doi:10.1016/j.mocell.2025.100263)
Supplement: Supplementary file 3 — Supplementary Figure [file mmc3.pdf]

Supplementary Figure 1.

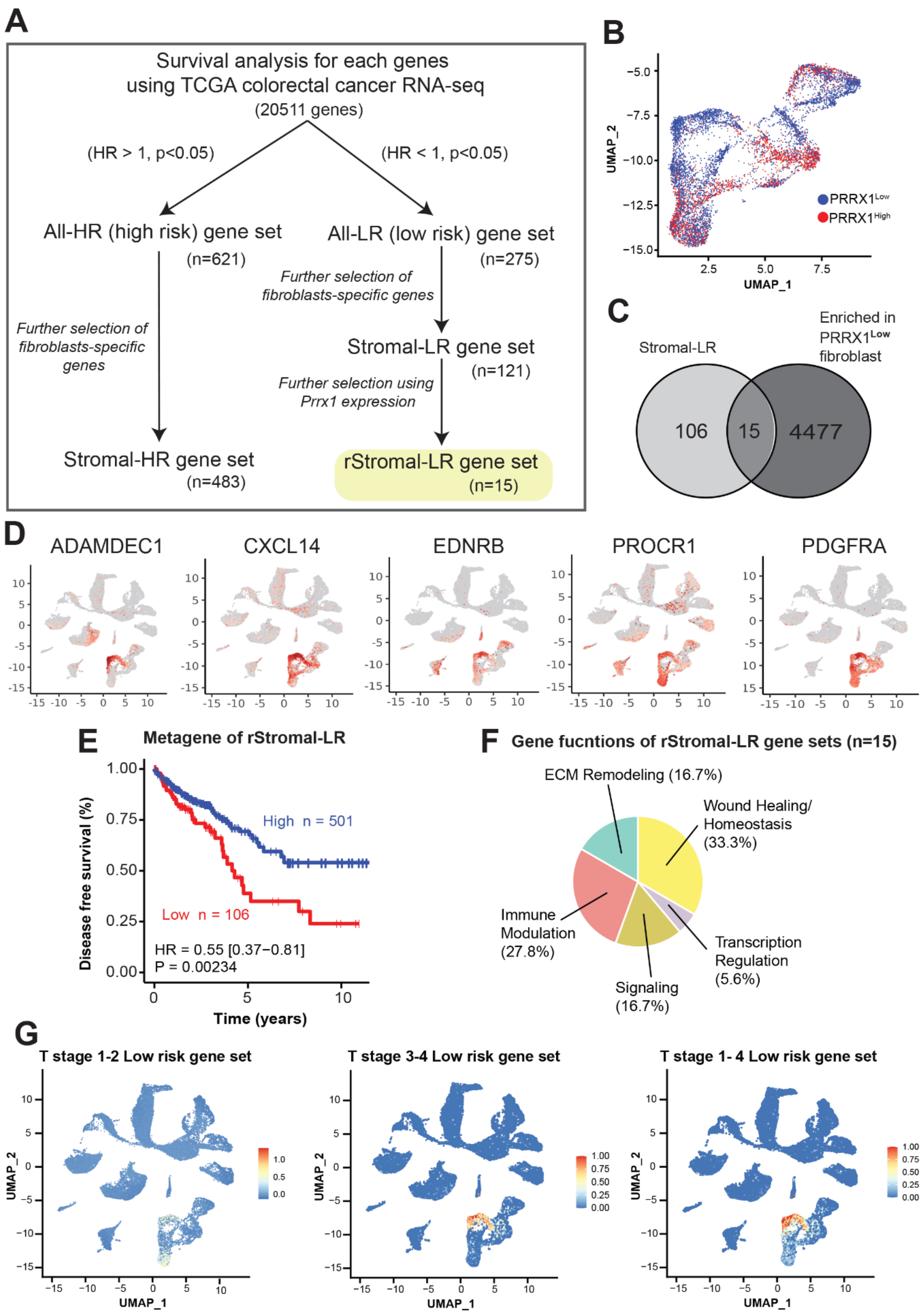

Supplementary Figure 1. Identification of a refined stromal low-risk gene set (rStromal-LR) associated with favorable prognosis.

(A) Pipeline for defining a refined fibroblast-specific low-risk gene set (termed rStromal-LR) by integrating scRNA-seq data (E-MTAB-8107) and TCGA survival data. A total of 20,511 genes identified from the scRNA-seq dataset were first classified into high-risk (HR) and low-risk (LR) groups based on TCGA survival analysis (HR > 1 or HR < 1, $p < 0.05$). Stromal-related genes were then extracted to define the Stromal-HR (n = 483) and Stromal-LR (n = 121) gene sets. Finally, a refined subset of Stromal-LR genes with enrichment in PRRX1^{low} subpopulation was selected, resulting in the final rStromal-LR gene set (n = 15). (B) UMAP plot showing fibroblasts with high (red) and low (blue) PRRX1 expression distributed across distinct regions. (C) Venn diagram showing the overlap between the Stromal-LR gene set and genes enriched in PRRX1^{low} fibroblasts. (D) Feature plots showing the expression of representative rStromal-LR genes (ADAMDEC1, CXCL14, EDNRB, PROCR1, and PDGFRA). (E) Kaplan–Meier disease-free survival analysis based on a metagene score (enrichment score) of the 15 rStromal-LR genes. Survival curves are shown for patient groups with high (n = 501) and low (n = 106) rStromal-LR gene expression (HR = 0.55, p = 0.00234). (F) Pie chart representing the functional categorization of rStromal-LR genes (n = 15), showing proportions involved in wound healing/homeostasis (33.3%), immune modulation (27.8%), ECM remodeling (16.7%), signaling (16.7%), and transcription regulation (5.6%). (G) UMAP projections of low-risk gene sets associated with reduced DFS in early (T stages 1–2), advanced (T stages 3–4), and pooled (T stages 1–4) colorectal tumors.

Supplementary Figure 2.

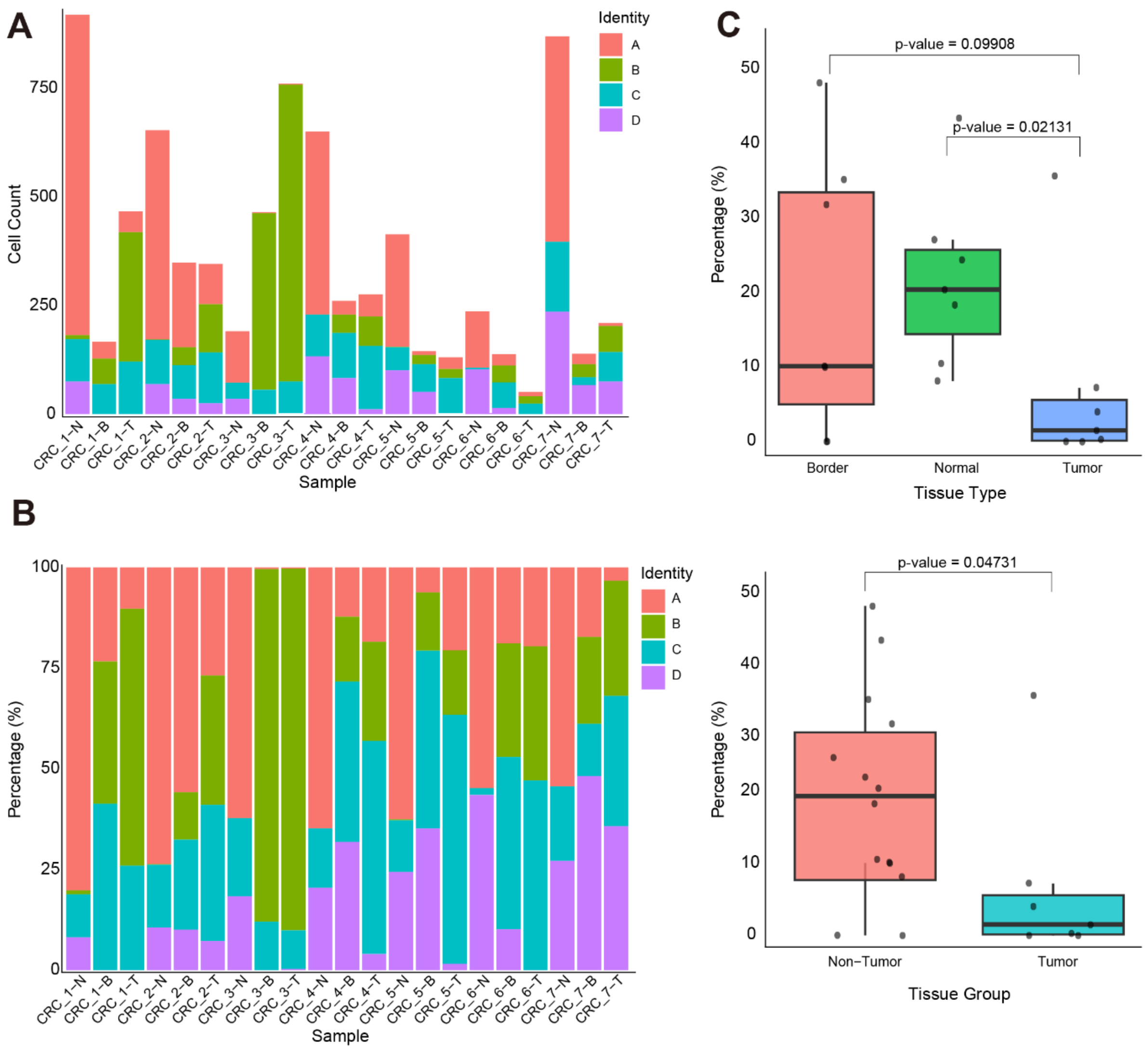

Supplementary Figure 2. Fibroblast cluster composition in individual samples from the E-MTAB-8107 dataset.

(A) Bar plot showing the absolute number of cells per cluster in each sample. (B) Bar plot showing the relative percentage of each cluster per sample. (C) Box plots comparing the proportion of Group D fibroblasts. The upper panel compares fibroblast composition across border, normal, and tumor tissues, while the lower panel compares non-tumor (normal + border) versus tumor tissues. Statistical analysis was performed using the Wilcoxon rank-sum (Mann–Whitney U) test.

Supplementary Figure 3

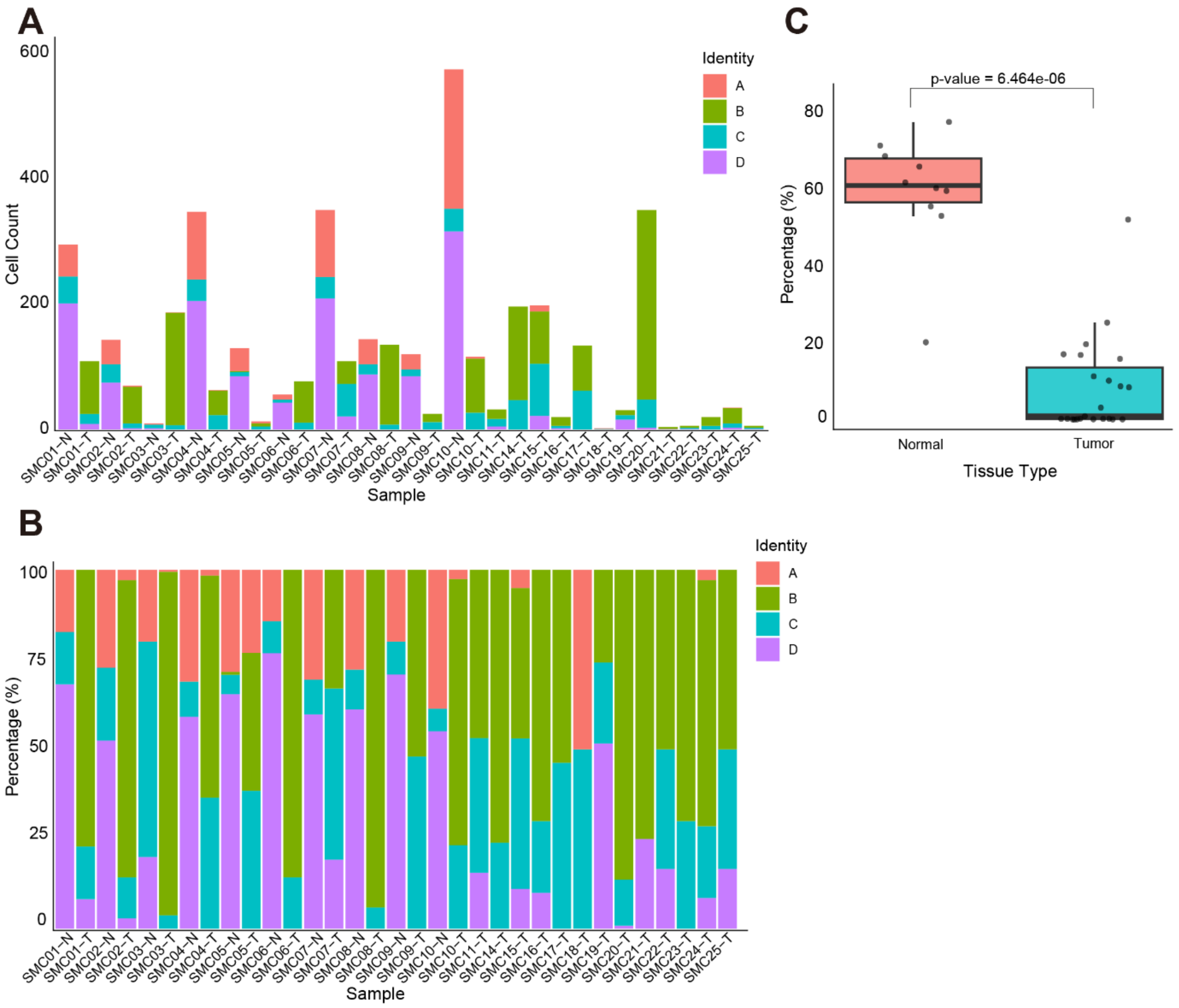

Supplementary Figure 3. Fibroblast cluster composition in individual samples from the GSE145686 dataset.

(A) Bar plot showing the absolute number of cells per cluster in each sample. (B) Bar plot showing the relative percentage of each cluster per sample. (C) Box plots compare the proportion of Group D fibroblasts between normal and tumor tissues. Statistical analysis was performed using the Mann–Whitney U test.

Supplementary Figure 4

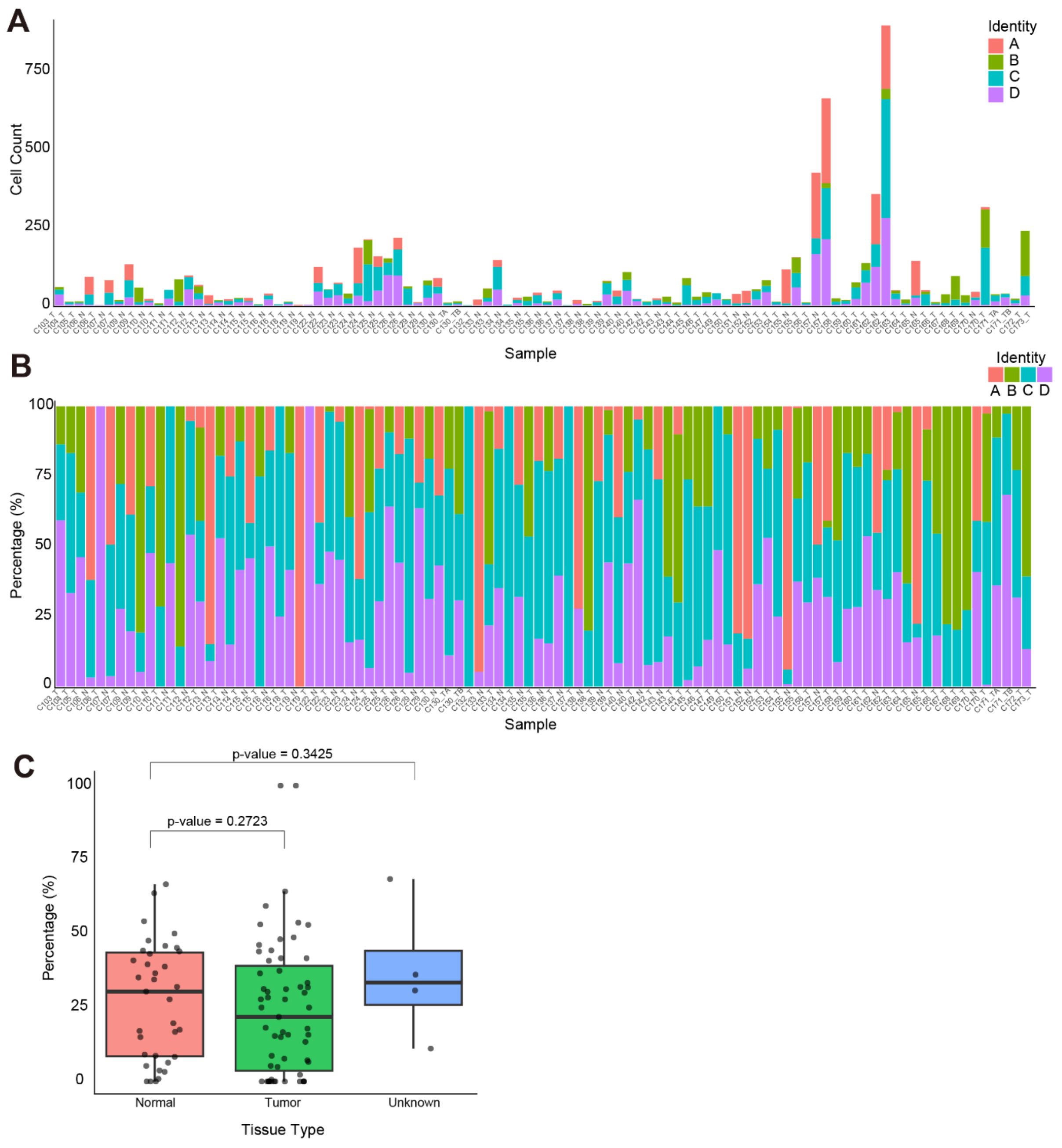

Supplementary Figure 4. Fibroblast cluster composition in individual samples from the GSE178341 dataset.

(A) Bar plot showing the absolute number of cells per cluster in each sample. (B) Bar plot showing the relative percentage of each cluster per sample. (C) Box plots compare the proportion of Group D fibroblasts between normal and tumor tissues. Statistical analysis was performed using the Wilcoxon rank-sum test.

Supplementary Figure 5

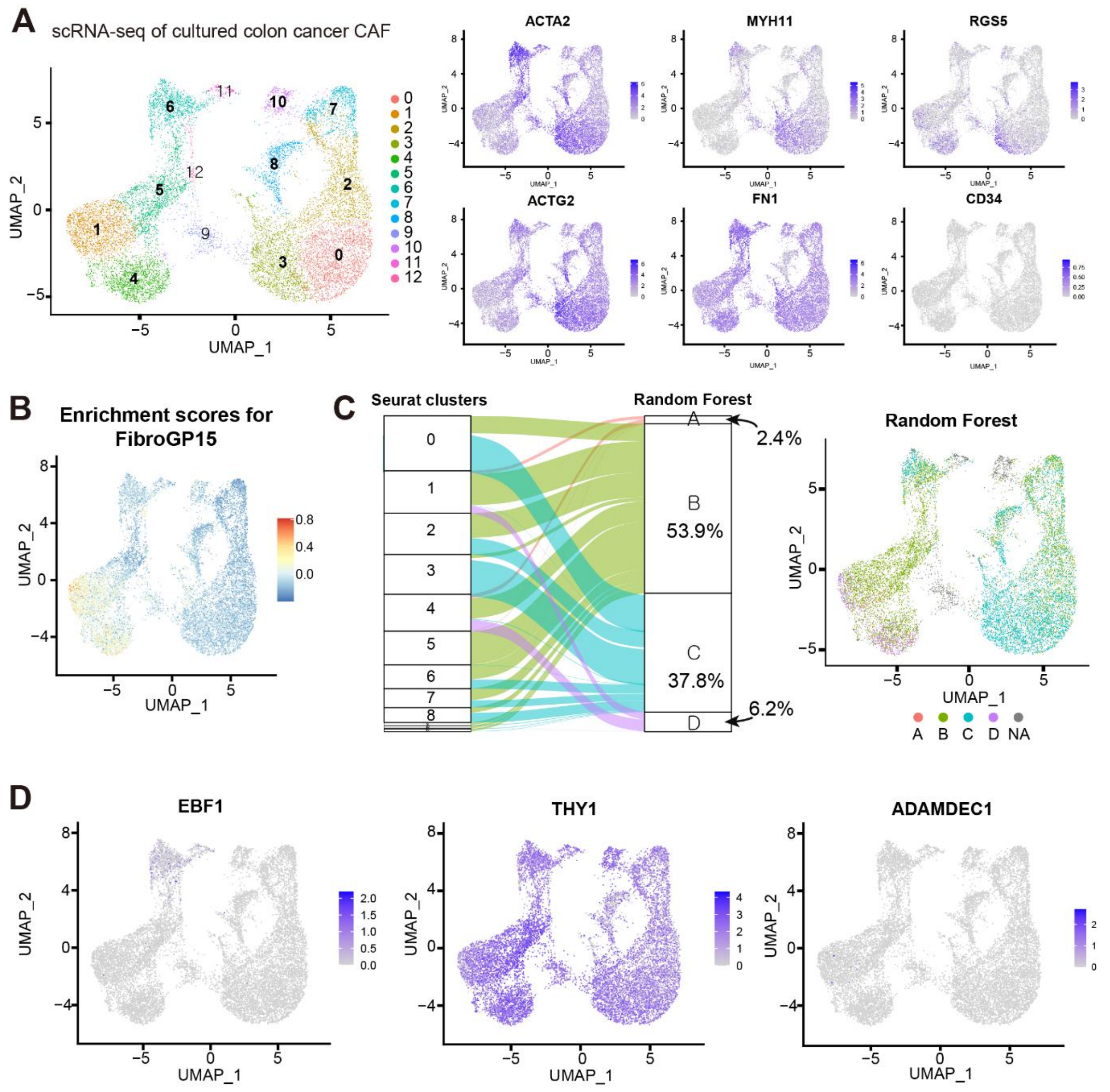

Supplementary Figure 5. Ex vivo single-cell analysis of fibroblast cultures reveals rStromal-LR **enrichment and the distribution of prognosis-associated fibroblast clusters.**

(A) scRNA-seq analysis of *ex vivo* fibroblast cultures from colon cancer tissue and expressions of major stromal cell markers. (B) Enrichment scores of rStromal-LR gene set in each cell of *ex vivo* fibroblast cultures. (C) Estimation of the proportions of fibroblast clusters such as A, B, C, and D in *ex vivo* fibroblast cultures using a random forest classifier. (D) The expression of crucial differentiation markers in *ex vivo* fibroblast cultures

Supplementary Figure 6.

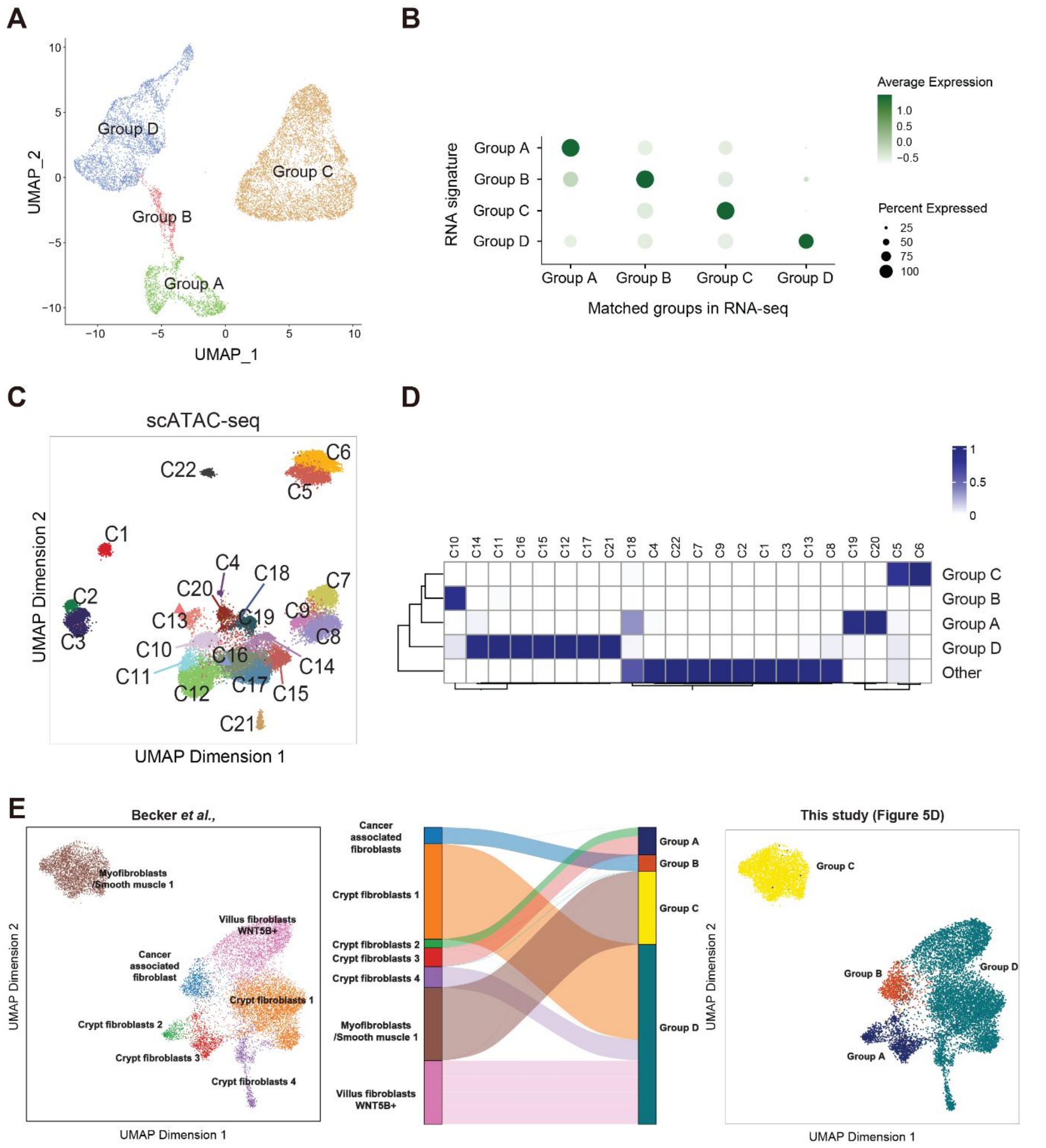

Supplementary Figure 6. Annotation and Comparative Analysis of Fibroblast Subpopulations in scRNA-seq and scATAC-seq Between Becker et al. and This Study

(A) UMAP projections of CAFs scRNA-seq from adjacent normal tissues and colorectal cancer patients. (B) Dot plot showing the AddModuleScores for marker genes specific to Groups A-D. (C) UMAP projections of scATAC-seq from stromal cells derived from adjacent normal tissues and colorectal cancer patients. (D) Heatmap depicting the activity levels of marker genes specific to Groups A-D. (E)

(Left) UMAP projection of fibroblast clusters as originally defined by Becker *et al.*, comprising seven subgroups: Crypt fibroblasts 1–4, Villus fibroblasts WNT5B+, Myofibroblasts/Smooth muscle cells, and Cancer-associated fibroblasts. (Center) Sankey diagram illustrating the correspondence between Becker *et al.*'s seven fibroblast clusters and the four transcriptionally coherent groups defined in this study (Groups A–D). (Right) UMAP projection of fibroblasts based on re-clustering of this study, showing functional re-classification into four major groups. Importantly, Crypt Fibroblasts 1 and 4, along with Villus Fibroblasts WNT5B+, are re-classified into Group D exhibiting tumor-restraining characteristics, based on pseudotime analysis, where Group D originates from Group A.

Supplementary Figure 7.

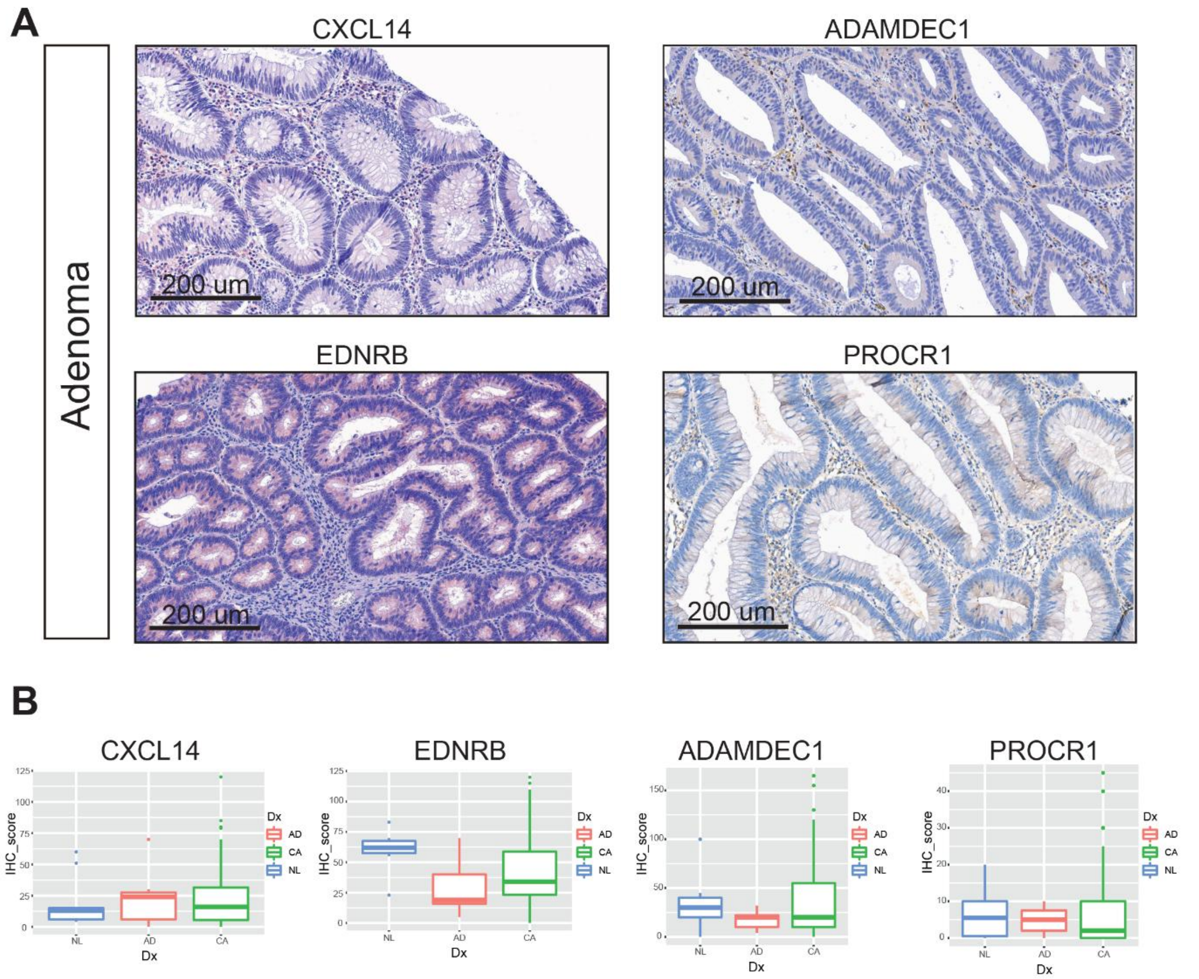

Supplementary Figure 7. Immunohistochemical Staining of Tr-CAF Markers in colon Adenoma and IHC Scores according to Diagnosis.

(A) Immunohistochemical staining was performed on adenoma samples for ADAMDEC1, CXCL14, PROCR, and EDNRB. (B) Box plot illustrating the IHC scores of each Tr-CAF marker based on different diagnose.

Supplementary Figure 8.

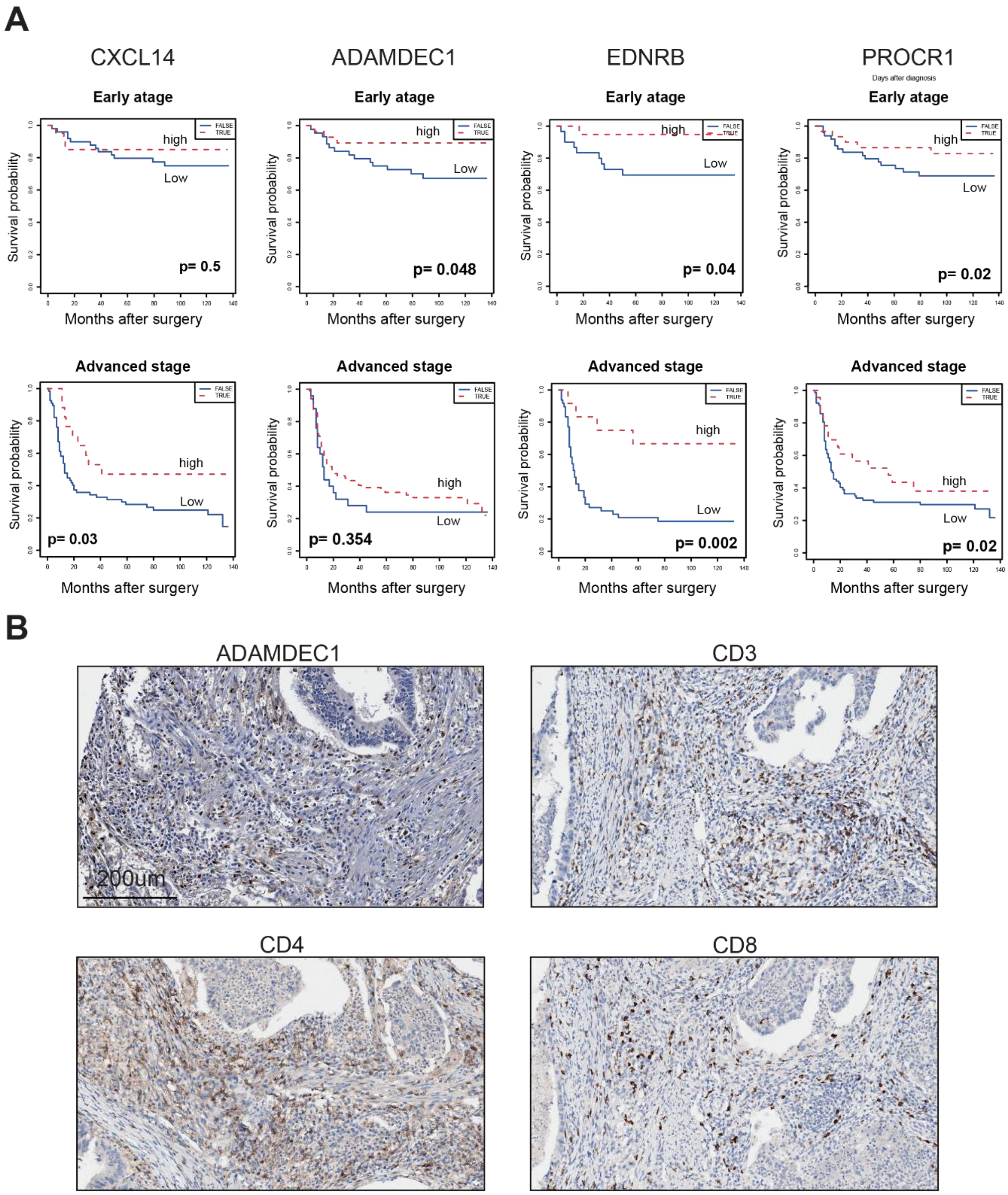

Supplementary Figure 8. Survival Rates of High Tr-CAF Marker-Expressing Patients and Correlation between ADAMDEC1 and T cells.

(A) Kaplan-Meier survival curves based on the expression levels of each Tr-CAF marker in Early and Advanced state. (B) Representative Immunohistochemistry (IHC) Image demonstrating elevated co-expression of CD3, CD4, and CD8 T-cell markers with high ADAMDEC1 levels in patient.
